# Discordant associations of educational attainment with ASD and ADHD implicate a polygenic form of pleiotropy

**DOI:** 10.1038/s41467-021-26755-1

**Published:** 2021-11-11

**Authors:** Ellen Verhoef, Jakob Grove, Chin Yang Shapland, Ditte Demontis, Stephen Burgess, Dheeraj Rai, Anders D. Børglum, Beate St Pourcain

**Affiliations:** 1 Language and Genetics Department, Max Planck Institute for Psycholinguistics, Nijmegen, The Netherlands; 2 International Max Planck Research School for Language Sciences, Nijmegen, The Netherlands; 3 The Lundbeck Foundation Initiative for Integrative Psychiatric Research, iPSYCH, Aarhus, Denmark; 4 Department of Biomedicine (Human Genetics) and Centre for Integrative Sequencing, iSEQ, Aarhus University, Aarhus, Denmark; 5 Center for Genomics and Personalized Medicine, Aarhus, Denmark; 6 Bioinformatics Research Centre, Aarhus University, Aarhus, Denmark; 7 MRC Integrative Epidemiology Unit, University of Bristol, Bristol, UK; 8 Population Health Sciences, University of Bristol, Bristol, UK; 9 MRC Biostatistics Unit, University of Cambridge, Cambridge, UK; 10 Cardiovascular Epidemiology Unit, Department of Public Health and Primary Care, University of Cambridge, Cambridge, UK; 11 Centre for Academic Mental Health, Bristol Medical School, University of Bristol, Bristol, UK; 12 NIHR Biomedical Research Centre, University of Bristol, Bristol, UK; 13 Avon and Wiltshire Partnership NHS Mental Health Trust, Bristol, UK; 14 Donders Institute for Brain, Cognition and Behaviour, Radboud University, Nijmegen, The Netherlands

**Keywords:** Computational biology and bioinformatics, Behavioural genetics, Neurodevelopmental disorders, Population genetics, Psychiatric disorders

## Abstract

Autism Spectrum Disorder (ASD) and Attention-Deficit/Hyperactivity Disorder (ADHD) are complex co-occurring neurodevelopmental conditions. Their genetic architectures reveal striking similarities but also differences, including strong, discordant polygenic associations with educational attainment (EA). To study genetic mechanisms that present as ASD-related positive and ADHD-related negative genetic correlations with EA, we carry out multivariable regression analyses using genome-wide summary statistics (*N* = 10,610–766,345). Our results show that EA-related genetic variation is shared across ASD and ADHD architectures, involving identical marker alleles. However, the polygenic association profile with EA, across shared marker alleles, is discordant for ASD versus ADHD risk, indicating independent effects. At the single-variant level, our results suggest either biological pleiotropy or co-localisation of different risk variants, implicating MIR19A/19B microRNA mechanisms. At the polygenic level, they point to a polygenic form of pleiotropy that contributes to the detectable genome-wide correlation between ASD and ADHD and is consistent with effect cancellation across EA-related regions.

## Abstract (web summary)

Autism Spectrum Disorder and Attention-Deficit/Hyperactivity Disorder are co-occurring neurodevelopmental conditions displaying strong, discordant polygenic associations with educational attainment. Here, the authors study genetic mechanisms underlying genome-wide correlation patterns across these traits.

## Introduction

Autism Spectrum Disorder (ASD) and Attention-Deficit/Hyperactivity Disorder (ADHD) are genetically complex childhood-onset neurodevelopmental conditions^1,2^ that often co-occur^3^. Approximately 15–25% of individuals with ADHD show ASD symptoms, and ~40–70% of individuals with ASD have a comorbid ADHD symptomatology^3^, although knowledge of shared aetiological mechanisms is scarce.

Like many other complex psychiatric disorders, ASD and ADHD are highly polygenic, and the majority of genetic influences can be attributed to common genetic variation^4^. There is increasing evidence from twin and genome-wide association studies (GWAS)^5,6^ suggesting genetic links between ASD and ADHD symptoms, both throughout population variation^7–13^ and at the clinical level^14^. The largest and most recent cross-disorder GWAS reported at least a hundred loci (as tagged by single variants) with pleiotropic effects on more than one disorder, including ASD and ADHD^4^. A model of single-nucleotide polymorphism (SNP)-based genetic correlations among multiple psychiatric disorders, using exploratory factor analyses and genomic structural equation models, showed that both ASD and ADHD are part of the same cluster of early-onset neurodevelopmental disorders^4^. The existence of genetic links between these disorders is further strengthened by the familial co-aggregation of both clinical disorders in large register-based studies^15^ and the identification of shared copy number variations, suggesting similar biological pathways^16^.

Estimates of genetic correlations between ASD and ADHD diagnosis range from 0.36(95%-confidence interval(CI): 0.26–0.46)^17^ in molecular studies to 0.87(95%-CI: 0.77–1.0)^18^ in twin analyses^19^. Evidence for genetic links between ASD and ADHD symptom co-occurrence can be even stronger in population-based samples^8^. However, when both, clinical ASD and clinical ADHD, are investigated with respect to a third genetically complex trait, some differences in the genetic architecture become apparent. Each disorder, when predicted with GWAS variants, reveals an opposite genetic correlation with cognitive functioning and educational attainment (EA). While increased polygenic ADHD risk has been linked to lower cognitive abilities and EA^20,21^, increased polygenic ASD risk has been associated with higher cognitive functionality and EA^17,20,22^. This discordant association pattern is most discernible for measures of years of schooling and college completion^17,21^. Observational research in ADHD strongly confirms the associations with lower school performance and educational outcomes^23^. Reports of academic achievement in ASD are more variable^24^, although high-functioning individuals can obtain higher-order qualifications, despite disadvantages in the labour market^25^.

**Fig. 1:**
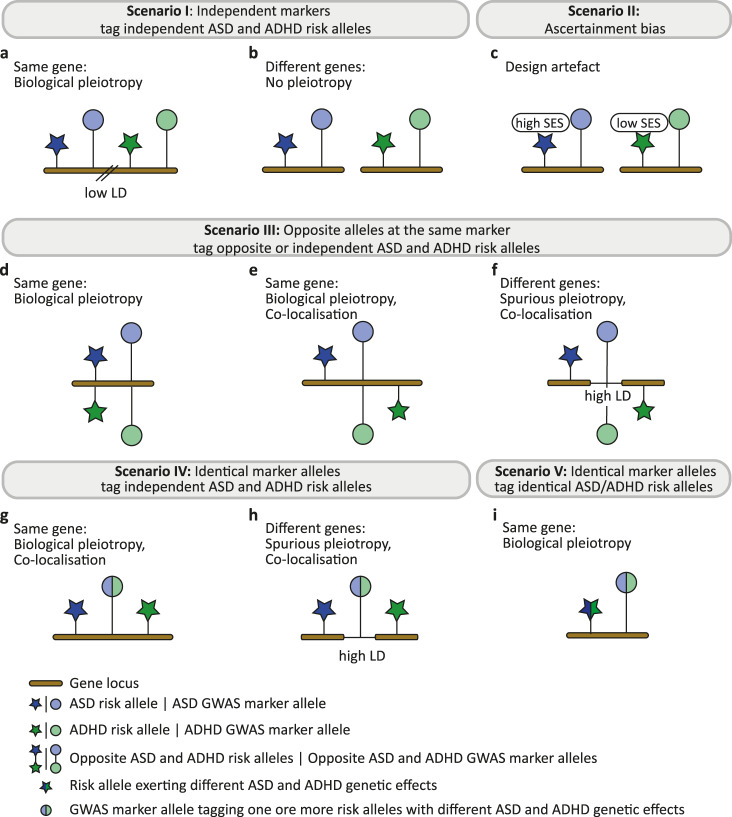
Candidate mechanisms underlying discordant genetic association patterns with educational attainment. Discordant associations with educational attainment, such as observed for ASD versus ADHD risk, may arise due to different mechanisms. Scenario I: Independent markers tag independent ASD and ADHD risk alleles, either (**a**) at the same gene locus in regions with low linkage disequilibrium (LD) or (**b**) at different loci. (**c**) Scenario II: Ascertainment bias during the recruitment of cases may lead to an artificial association of ASD with higher socio-economic status (SES) and ADHD with lower SES (non-testable). Scenario III: Opposite alleles at the same marker may tag opposite ASD and ADHD risk alleles at (**d**) a single risk variant, or independent ASD and ADHD risk alleles within (**e**) the same gene or (**f**) different genes in high-LD regions. Scenario IV: Identical marker alleles tag independent ASD and ADHD risk alleles, either (**g**) within the same gene or (**h**) at different genes in high-LD regions. (**i**) Scenario V: Identical marker alleles tag identical ASD/ADHD risk alleles (biological pleiotropy). Within each subfigure, one or more observed marker allele is shown in linkage disequilibrium with one or more ASD and ADHD risk allele. ADHD Attention-Deficit/Hyperactivity Disorder, ASD Autism Spectrum Disorder, GWAS genome-wide association study, LD, linkage disequilibrium, SES socio-economic status.

The mechanisms underlying the discordant polygenic association pattern with EA are not yet known and may involve different biological effects, including pleiotropy. Following Solofiev and colleagues^26^, we define biological pleiotropy as processes where the same gene has a direct biological influence on more than one phenotype. In contrast, spurious pleiotropy involves multiple sources of bias that cause a false association between a gene and multiple phenotypes^26^. Different causal risk variants in high linkage disequilibrium (LD) with the same marker are described as co-localising variants^26^. We do not consider mechanisms of mediated pleiotropy, i.e. an indirect association between a genetic variant and a further phenotype that arises due to causal associations between phenotypes^26^, as this mechanism would imply concordant associations between EA and both ASD and ADHD risk. An overview of candidate mechanisms underlying discordant genetic associations is shown in Fig. 1.

First (scenario I), the set of underlying causal variants linking ASD to EA might be independent of the set of causal variants linking ADHD to EA. Here, independent GWAS marker alleles may tag independent ASD and ADHD risk alleles, either residing within regions of low LD at the same gene locus (biological pleiotropy, Fig. 1a) or at different loci (no pleiotropy, Fig. 1b). Second (scenario II), discordant genetic association patterns with EA may arise because of ascertainment bias during the recruitment of ASD and ADHD cases (Fig. 1c). In the US, the prevalence of ASD has been associated with higher parental socio-economic status (SES)^27^. In a large population-based study in Sweden, where free ASD services are available to all regardless of SES, an association between lower SES and higher ASD risk^28^ has been observed. In contrast, children in low SES families are consistently more likely to receive a diagnosis of ADHD than children in high SES families^29^. Third (scenario III), opposite GWAS marker alleles may tag opposite causal ASD and ADHD risk alleles at the same risk variant (biological pleiotropy, Fig. 1d), or they may tag independent ASD and ADHD risk alleles, either within the same gene (biological pleiotropy, co-localising variants, Fig. 1e), or at different loci in high LD (spurious pleiotropy, co-localising variants, Fig. 1f). The most recent GWAS across multiple psychiatric disorders identified several loci with opposite directional allelic effects at the 10^−6^ *P*-value threshold^4^. None of these loci were shared between ASD and ADHD^4^ though these effects may become more prevalent when applying less stringent GWAS marker selection criteria. Fourth (scenario IV), identical GWAS marker alleles may tag independent ASD and ADHD risk alleles due to high LD^26^, either at the same locus (biological pleiotropy, co-localising variants, Fig. 1g) or at different loci (spurious pleiotropy, co-localising variants, Fig. 1h). The vast majority of trait-associated loci across the genome is associated with multiple traits and each physical location can contain multiple groups of variants with independent genetic effects^30,31^. Hence, the distribution of genetic effects across the same GWAS marker alleles might differ for ASD and ADHD risk and, consequently, shape polygenic associations with a third phenotype, such as EA. Finally (scenario V), risk alleles might be shared across ASD and ADHD liability but exert, because of biological pleiotropy, different genetic effects. Here, different single-variant genetic effects may lead to different polygenic associations with EA (Fig. 1i), as captured by identical GWAS marker alleles. Note that in the absence of pleiotropy, identical ASD and ADHD risk alleles would lead to a concordant and not a discordant polygenic association with EA.

**Fig. 2:**
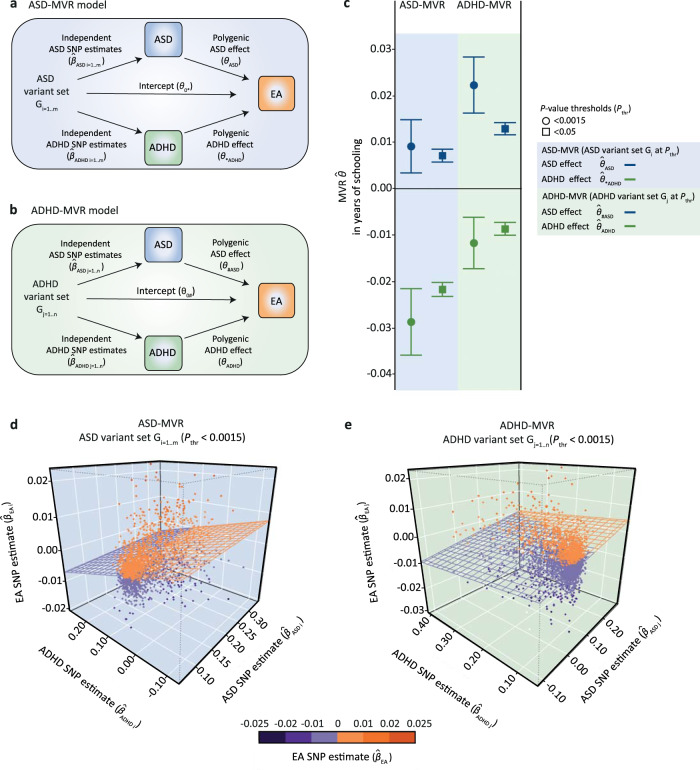
Multivariable regression models. Acyclic graphs illustrating the multivariable regression (MVR) design for (**a**) a set of independent ASD-related variants G_i_ (ASD-MVR) and (**b**) a set of independent ADHD-related variants G_j_ (ADHD-MVR). For both MVR models, increaser marker alleles (G_i_ or G_j_) were aligned to ASD ($\hat{\beta }$_ASD_), ADHD ($\hat{\beta }$_ADHD_) and EA ($\hat{\beta }$_EA_) GWAS SNP estimates, capturing genetic association at the single-variant level. Using a bidirectional MVR framework (ASD-MVR; ADHD-MVR), the aggregate association effect with EA across all variants was simultaneously estimated for ASD risk ($\theta$_ASD_; $\theta$_#ASD_) and ADHD risk ($\theta$_ADHD_; $\theta$_*ADHD_), including a regression intercept ($\theta$_0*_; $\theta$_0#_). *Estimation of polygenic risk effects using alleles that were selected to increase liability for ASD, but are shared, by position, with ADHD risk. ^#^Estimation of polygenic risk effects using alleles that were selected to increase liability for ADHD, but are shared, by position, with ASD risk. (**c**) ASD-MVR ($\hat{\theta }$_ASD_; $\hat{\theta }$_*ADHD_) and ADHD-MVR ($\hat{\theta }$_ADHD_; $\hat{\theta }$_#ASD_) effects as change in years of schooling per increase in log odds of ASD or ADHD liability. Multivariate inverse-variance-weighted regression estimates and corresponding 95% confidence intervals (bars) are shown. Individual effect estimates, standard errors and corresponding *P*-values (*t*-statistic, two-sided test) are provided in Supplementary Table 3. All tests passed the multiple-testing threshold of *P* < 0.0023. G_i_ was selected from ASD(iPSYCH, woADHD) and G_j_ from ADHD(iPSYCH), both at *P*_thr_ < 0.0015 and *P*_thr_ < 0.05. ASD ($\hat{\beta }$_ASD_), ADHD ($\hat{\beta }$_ADHD_) and EA ($\hat{\beta }$_EA_) SNP estimates were extracted from ASD(iPSYCH, woADHD; *N* = 32,985), ADHD(iPSYCH; *N* = 37,076) and EA(SSGAC; *N* = 766,345) GWAS statistics respectively. 3D scatter plot of (**d**) ASD-MVR (G_i_; *P*_thr_ < 0.0015) and (**e**) ADHD-MVR (G_j_; *P*_thr_ < 0.0015), as shown in (**c**). The regression plane reflects the estimated MVR effects for ASD-MVR ($\hat{\theta }$_ASD_; $\hat{\theta }$_*ADHD_) and ADHD-MVR ($\hat{\theta }$_ADHD_; $\hat{\theta }$_#ASD_), respectively. Source data are provided as a Source data file. ADHD Attention-Deficit/Hyperactivity Disorder, ASD Autism Spectrum Disorder, EA educational attainment, MVR multivariable regression, *P*_thr_, *P*-value threshold, SNP single-nucleotide polymorphism.

In this work, we (i) study evidence for genetic mechanisms presenting as discordant polygenic association pattern with EA, specifically with respect to ASD and ADHD risk, and (ii) identify and annotate underlying ASD and ADHD risk variants. We model polygenic relationships between ASD, ADHD and EA using a multivariable regression (MVR) technique, a multivariate methodology based on summary statistics adopted from a causal modelling approach^32^. Without making causal inferences, as we allow for biological pleiotropy, this method can simultaneously estimate polygenic ASD and ADHD association with EA, while controlling for bias that may arise when adjusting for heritable covariates^33^. Specifically, we model polygenic links with EA as aggregate effects of independent genetic variants, and, subsequently, compare the direction of effect at the polygenic and single-marker level. The selection of genetic variant sets follows guidelines established for polygenic scoring methods^34^, without generating accumulated risk allele scores. To this end, we study SNP estimates from existing GWAS summary statistics for EA, ASD and ADHD (Table 1) using a bidirectional MVR approach (Fig. 2) and assess evidence in support of genetic mechanisms as outlined in Fig. 1, except for hidden ascertainment bias (scenario II). Here, we show that EA-related genetic variation is shared across ASD and ADHD architectures, involving identical alleles at the same markers. Despite positive genetic correlations at the single-variant level, these shared marker alleles contribute to discordant and, thus, independent polygenic associations of EA with ASD versus ADHD risk.

**Table 1:** Sample description.

Source	Phenotype	Consortium	GWAS	Imputation reference panel	*N*
Clinical sample	ASD	iPSYCH	ASD(iPSYCH,woADHD)^17^	1000 Genomes phase 3	32,985 (10,321 cases)
Clinical sample	ASD	PGC	ASD(PGC)^35^	1000 Genomes phase 1 (v3)	10,610 (5,305 cases)
Clinical sample	ADHD	iPSYCH	ADHD(iPSYCH)^21^	1000 Genomes phase 3	37,076 (14,584 cases)
Population sample	Years of schooling	SSGAC	EA(SSGAC)^44^	1000 Genomes phase 3^a^	766,345

All individuals were of European descent.

*ADHD* Attention-Deficit/Hyperactivity Disorder, *ASD* Autism Spectrum Disorder, *EA* educational attainment, *iPSYCH* The Lundbeck Foundation Initiative for Integrative Psychiatric Research, *PGC* Psychiatric Genomics Consortium, *SSGAC* Social Science Genetic Consortium, *woADHD* without ADHD.

^a^Predominantly 1000 genomes phase 3, see Lee et al.^44^.

## Results

### Identification of genetic mechanisms underlying discordant polygenic association patterns with educational attainment

We investigated polygenic relationships between ASD, ADHD and EA with a weighted MVR framework (‘Methods’, Formulae 1–4), analogous to Mendelian randomization (MR) approaches^32^, studying sets of independent subthreshold GWAS markers as selected for polygenic scoring analyses^34^. Given that disorder-related GWAS markers can be identified with respect to both ASD and ADHD risk, we adopted a bidirectional approach with two complementary MVR designs:

1. For an ASD-MVR model (Fig. 2a), we selected a set of independent subthreshold variants from ASD GWAS summary statistics, G_i_ (with i = 1, …, m SNPs), across different *P*-value selection thresholds. Each ASD-increasing marker allele was aligned to an ASD ($\hat{\beta }$_ASD_), ADHD ($\hat{\beta }$_ADHD_) and EA ($\hat{\beta }$_EA_) SNP estimate, and the aggregate association effect with EA across all alleles was simultaneously estimated for ASD ($\hat{\theta }$_ASD_) and ADHD ($\hat{\theta }$_*ADHD_) risk. The “*” symbol indicates the estimation of a polygenic risk effect with EA, here ($\hat{\theta }$_*ADHD_), using alleles that were selected to increase liability for ASD, but are shared by position, here with ADHD risk.
2. For an analogous ADHD-MVR model (Fig. 2b), we used a set of independent subthreshold variants from an ADHD GWAS summary statistics, G_j_ (with j = 1, …, n SNPs), also generated across different *P*-value selection thresholds. Similarly, each ADHD-increasing marker allele was aligned to an ASD ($\hat{\beta }$_ASD_), ADHD ($\hat{\beta }$_ADHD_) and EA ($\hat{\beta }$_EA_) SNP estimate and their joint association with EA was simultaneously estimated for ASD ($\hat{\theta }$_#ASD_) and ADHD risk ($\hat{\theta }$_ADHD_). The ‘#’ symbol indicates the estimation of a polygenic risk effect with EA, here ($\hat{\theta }$_#ASD_), using alleles that were selected to increase liability for ADHD, but are shared by position, here, with ASD risk.

ASD- and ADHD-MVR models, and modifications thereof, were implemented in multiple stages of the study (Supplementary Fig. 1). ASD ($\hat{\beta }$_ASD_), ADHD ($\hat{\beta }$_ADHD_) and EA ($\hat{\beta }$_EA_) SNP estimates were extracted from GWAS summary statistics based on non-overlapping samples or cases, as provided by large consortia (Table 1).

As part of discovery analyses (Supplementary Fig. 1a–b), we first investigated whether discordant association patterns with EA involve independent markers that tag independent ASD and ADHD risk alleles (scenario I, Fig. 1a, b) or identical markers (scenario III–V, Fig. 1d–i). For this, we analysed a series of ASD-MVR (Fig. 2a) and ADHD-MVR models (Fig. 2b) using ASD (G_i_) and ADHD (G_j_) variants sets as selected from ASD(iPSYCH,woADHD) and ADHD(iPSYCH) GWAS summary statistics, respectively (11 *P*-value selection thresholds: 5 × 10^−8^ < *P*_thr_ < 0.5; multiple-testing-adjusted significance threshold: 0.0023 (0.05/22); shown for simplicity at *P*_thr_ < 0.05 and *P*_thr_ < 0.0015). These bidirectional MVR analyses, based on SNP estimates from ASD(iPSYCH,woADHD) and ADHD(iPSYCH) and EA(SSGAC) summary statistics, showed that inverse polygenic associations with EA can be captured for both, ASD and ADHD risk, using the same set of genetic variants, irrespective of the selection of G_i_ and G_j_ variants sets (Supplementary Tables 1–2). For example, for ASD-MVR (G_i_: *P*_thr_ < 0.0015, N_SNPs_ = 1,973, Fig. 2c, d, Supplementary Table 3), we observed evidence for a positive ASD association with EA, with an 0.009 increase in years of schooling per log-odds in ASD liability (ASD-MVR $\hat{\theta }$_ASD_ = 0.009 (SE = 0.003), *P* = 0.002). Simultaneously, the same ASD-related risk alleles captured a negative association between ADHD and EA with a 0.029 decrease in years of schooling per log odds in ADHD liability (ASD-MVR $\hat{\theta }$_*ADHD_ = −0.029 (SE = 0.004), *P* < 1 × 10^−10^). ADHD-MVR findings revealed a complementary association profile (Fig. 2). For ADHD-MVR (G_j_: *P*_thr_ < 0.0015, N_SNPs_ = 2,717), this corresponds to an 0.012 decrease in years of schooling per log odds in ADHD liability (ADHD-MVR $\hat{\theta }$_ADHD_ = −0.012(SE = 0.003), *P* = 4 × 10^−5^), and an increase in 0.022 years of schooling per log odds in ASD liability (ADHD-MVR $\hat{\theta }$_#ASD_ = 0.022(SE = 0.003), *P* < 1 × 10^−10^) (Fig. 2c, e, Supplementary Table 3).

Increasing the number of variants in G_i_ and G_j_ using more relaxed selection criteria (e.g. *P*_thr_ < 0.05) boosted the statistical power (Fig. 2c, Supplementary Table 1–3). Compared to univariable regression models (see ‘Methods’), the simultaneous estimation of polygenic ASD and ADHD effects on EA improved the model fit: multivariable models explained up to 3% more variation in genetically predictable EA, with only modest evidence for multi-collinearity (Supplementary Table 3, variance inflation factor (VIF) ≤ 1.2). Hence, the identification of discordant EA-related association profiles for ASD and ADHD risk captured by the same set of genetic markers is inconsistent with mechanisms involving independent ASD and ADHD genetic markers (scenario I, Fig. 1a, b).

Shared EA-related genetic variation across ASD and ADHD genetic architectures could be encoded via opposite ASD and ADHD risk alleles (scenario III, Fig. 1d–f), or identical ASD and ADHD risk alleles at the same GWAS marker (scenario IV/V, Fig. 1g–i). Within a next step, we therefore restricted discovery ASD (G_i_) and ADHD (G_j_) variant sets to markers carrying the same risk-increasing allele for both disorders (termed here forth concordant variants; ~80% of discovery G_i_ and G_j_ sets at *P*_thr_ < 0.0015 and *P*_thr_ < 0.05). MVR analyses with concordant variants confirmed the robustness of identified MVR effects, with little evidence for attenuation (multiple-testing-adjusted significance threshold: 0.0023, Supplementary Table 4). The corresponding bivariate relationships between SNP estimates for ASD, ADHD and EA (Supplementary Fig. 2) illustrate the positive correlation between ASD ($\hat{\beta }$_ASD_) and ADHD ($\hat{\beta }$_ADHD_) risk allele effects, but their inverse association with EA ($\hat{\beta }$_EA_), consistent with a positive LDSC genetic correlation between both disorders (Supplementary Table 5), and opposite genetic correlations with EA (Supplementary Table 6). Thus, discordant polygenic association with EA can be independently encoded across a set of shared ASD- and ADHD-related marker alleles. These findings are inconsistent with scenario III (Fig. 1d–f), implicating opposite alleles at the same marker. Instead, they suggest that identical marker alleles either tag independent ASD and ADHD risk alleles (scenario IV, Fig. 1g–h) or that they tag identical ASD/ADHD risk alleles that exert different effects due to biological pleiotropy (scenario V, Fig. 1i).

**Fig. 3:**
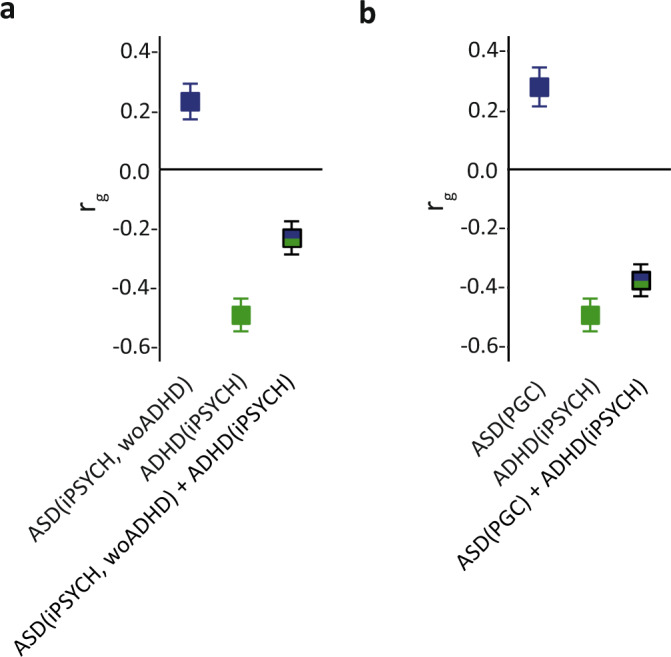
Genetic correlations with educational attainment for ASD + ADHD cross-disorder meta-analyses. Genetic correlations (*r*_g_) of educational attainment (EA) with ASD, ADHD and combined ASD + ADHD risk were estimated using unconstrained Linkage Disequilibrium Score correlation^67^. Genetic correlations with EA(SSGAC; *N* = 766,345) were estimated for (**a**) ASD(iPSYCH, woADHD; *N* = 32,985), ADHD(iPSYCH; *N* = 37,076) and a combination of these summary statistics (cross-disorder meta-analysis), and, analogously, for (**b**) ASD(PGC; *N* = 10,610), ADHD(iPSYCH, *N* = 37,076) and a combination of these summary statistics (cross-disorder meta-analysis). Cross-disorder meta-analyses were conducted with METACARPA, allowing for sample overlap^61^. LDSC correlation estimates with 95% confidence intervals (bars) are shown. Individual genetic correlation estimates, standard errors and corresponding *P*-values (Z-statistic, two-sided test) are provided in Supplementary Table 6. All tests passed the multiple-testing threshold of *P* < 0.002. Source data are provided as a Source data file. ADHD Attention-Deficit/Hhyperactivity Disorder, ASD Autism Spectrum Disorder, iPSYCH The Lundbeck Foundation Initiative for Integrative Psychiatric Research, PGC psychiatric genetics consortium, *r*_g_ genetic correlation, SSGAC Social Science Genetic Association Consortium, woADHD without ADHD.

Next, we followed up MVR discovery findings by replacing ASD SNP estimates ($\hat{\beta }$_ASD_) from ASD(iPSYCH,woADHD) with SNP estimates from the independent ASD(PGC) sample (Supplementary Fig. 1c, d). We repeated ASD-MVRs and ADHD-MVRs with the same sets of variants as studied in the discovery MVR analyses (G_i_ and G_j_ at *P*_thr_ < 0.0015 and *P*_thr_ < 0.05, multiple-testing-adjusted significance threshold: 0.0125 (0.05/4)) and confirmed the discordant genetic association pattern with EA at *P*_thr_ < 0.05 (Supplementary Table 7). Here, using ADHD-MVR, ADHD-related risk alleles (G_j_, *P*_thr_ < 0.05) captured a positive association between ASD and EA ($\hat{\theta }$_#ASD_ = 0.003(SE = 4 × 10^−4^), *P* < 1 × 10^−10^), despite zero genetic correlations between ADHD(iPSYCH) and ASD(PGC)(Supplementary Table 5). As a validation step, we confirmed the positive association of polygenic ASD risk with EA using ASD(PGC) SNP estimates (G_i_ at *P*_thr_ < 0.05: $\hat{\theta }$_ASD_ = 0.005(SE = 0.001), *P* < 1 × 10^−10^, G_i_ at *P*_thr_ < 0.0015: $\hat{\theta }$_ASD_ = 0.01(SE = 0.003), *P* = 0.003). Association patterns remained largely unchanged when the analyses were repeated with concordant variant sets, confirming the robustness of our findings (multiple-testing-adjusted significance threshold: 0.0125, Supplementary Table 8). The attenuation of signal, compared to discovery MVR findings, is consistent with the limited power of ASD(PGC)^35^ and the smaller number of aligned risk alleles across ADHD and ASD using the ASD(PGC)(~50%) compared to the ASD(iPSYCH,woADHD)(~80%) sample (Supplementary Note 1). Thus, our findings, validated by the use of different GWAS summary statistics, suggest that discordant, and thus independent, genetic association patterns with EA are encoded across identical GWAS marker alleles that carry either subthreshold ASD or ADHD risk. A combination of ASD and ADHD risk effects across these identical ASD and ADHD marker alleles may therefore lead to the cancellation of polygenic association effects with EA. This is consistent with the substantially decreased genetic correlation with EA for both, a combined ASD(iPSYCH,woADHD) + ADHD(iPSYCH) sample, and a combined ASD(PGC) + ADHD(iPSYCH) sample, when meta-analysing respective summary statistics while allowing for sample overlap (Fig. 3).

### Identification of high-confidence genomic regions

To identify genomic regions that contribute to discordant association patterns with EA, we applied the gwas-pw method^36^. Dividing the genome into approximately independent LD blocks, this method estimates the posterior probability that a given genomic region contains shared or non-shared genetic effects for two complex traits, while correcting for sample overlap^36^. In particular, gwas-pw can identify LD blocks carrying genetic markers associated with both ASD and ADHD risk due biological pleiotropy (scenario V, Fig. 1i) or high-LD co-localisation (scenario IV, Fig. 1g, h), and distinguish them from LD blocks carrying multiple genetic markers that are each associated with a different disorder (co-localisation in the presence of low/moderate LD)^36^. Analysing ASD(iPSYCH,woADHD) and ADHD(iPSYCH) statistics, we identified evidence for shared genetic effects assuming a model of biological pleiotropy/high-LD co-localisation at three independent genomic regions (posterior probability > 0.9, Supplementary Fig. 3a). Each of these LD blocks harbours genes that have previously been linked to either ASD or ADHD risk at the genome-wide or suggestive level^17,21^, or, potentially, involve single-variant co-localisation^37^: chromosome 1p21.3 (1,734 kb: *PTBP2*^17^, *PTBPLP*, *7SK*, *DPYD*^37^, *DPYD-AS1*^37^), chromosome 5q14.3 (1,500 kb: *TMEM161B*^21^, *TMEM161B-AS1*^21^, *LINC00461*^21^, *MIR9-2*^21^, *MEF2C*^21^, *AL050132*) and chromosome 20p11.22-23 (1524 kb: *PLK1S1*(*KIZ*)^17^, *BC042893*, *BC034426*, *XRN2*^17^, *NKX2-2*, *NKX2-4*^17^, *Nkx2_2as*, *PAX1*^37^, *LOC100270679*, *CR627206*, *LOC284788*) (Supplementary Fig. 4). Given the polygenic nature of our findings, we selected independent ASD-related (N_SNPs_ = 465) and ADHD-related (N_SNPs_ = 481) variants from each LD block (LD-r^2^ < 0.25 within ±500 kb), beyond the assumption of a single causal locus, and repeated ASD-MVR and ADHD-MVR models without applying a *P*-value threshold (Supplementary Fig. 1e, f). The analyses across the three LD regions largely confirmed the discordant polygenic association of ASD and ADHD risk with EA (Supplementary Table 9). However, compared to discovery analyses (Supplementary Table 3), MVR effects were weaker and the polygenic ASD effect as estimated with ASD-MVR only passed the nominal significance level (multiple-testing-adjusted significance threshold: 0.0023, Supplementary Table 9). Within variant-based gwas-pw analyses, the strongest signal was observed for rs4916723 (posterior probability of 0.78), residing on chromosome 5, but did not pass stringent identification criteria (i.e. a posterior probability of >0.9). It is possible that, given the conservative correction of sample overlap, gwas-pw is partialling out also genuine polygenetic correlations between disorders^36^. Thus, the number of subthreshold variants and genomic regions contributing to discordant association patterns with EA might be considerably larger and, consequently, the power of identifying variants of small effects decreased.

Carrying out gwas-pw with ASD(PGC) and ADHD(iPSYCH) GWAS summary statistics did not result in the identification of genomic regions with pleiotropic/high-LD co-localisation signals (posterior probability <0.5, Supplementary Fig. 3b), largely reflecting the low power of the ASD(PGC) sample (posterior probability for ASD effects <0.1, Supplementary Fig. 3b).

### Identification of single variants

**Fig. 4:**
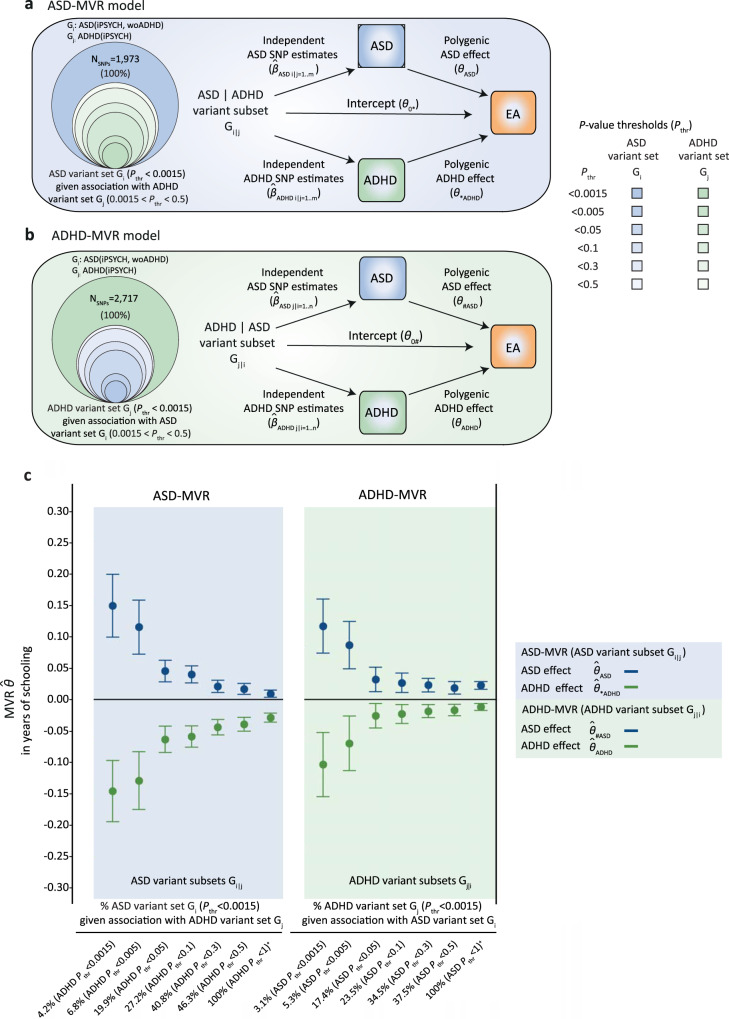
Identification of single variants using conditional *P*-value thresholding. Acyclic graphs illustrating the multivariable regression (MVR) design for (**a**) a set of independent ASD-related variants G_i_, given joint association with ADHD variant set G_j_ (ASD-MVR with G_i|j_) and (**b**) a set of independent ADHD-related variants G_j_, given joint association with ASD variant set G_i_ (ADHD-MVR with G_j|i_). Here, G_i|j_ and G_j|i_ are illustrated as subsets (concentric circles) of G_i_ and G_j_ across a grid of six *P*-values thresholds (0.0015 < *P*_thr_ *<* 0.5), based on ASD(iPSYCH, woADHD) and ADHD(iPSYCH) summary statistics. (**c**) ASD-MVR ($\hat{\theta }$_ASD_; $\hat{\theta }$_*ADHD_) and ADHD-MVR ($\hat{\theta }$_ADHD_; $\hat{\theta }$_#ASD_) effects as change in years of schooling per increase in log odds of ASD or ADHD liability (for the definition of $\hat{\theta }$_*ADHD_ and $\hat{\theta }$_#ASD_, see Fig. 2). SNP sets G_i|j_ and G_j|i_ were selected from ASD(iPSYCH, woADHD) and ADHD(iPSYCH), as shown in (a, b). SNP estimates for ASD ($\hat{\beta }$_ASD_), ADHD ($\hat{\beta }$_ADHD_) and EA ($\hat{\beta }$_EA_) were extracted from ASD(iPSYCH,woADHD; *N* = 32,985), ADHD(iPSYCH; *N* = 37,076) and EA(SSGAC; *N* = 766,345) GWAS statistics respectively. Multivariate inverse-variance-weighted regression estimates and corresponding 95% confidence intervals (bars) are shown. Individual effect estimates, standard errors and corresponding *P*-values (*t*-statistic, two-sided test) are provided in Supplementary Tables 10–11. All MVR effects passed the multiple-testing threshold of *P* *<* 0.0023, except for ADHD effects estimated with ADHD-MVR (G_j|i_: ADHD *P*_thr_ *<* 0.0015; ASD *P*_thr_ *<* 0.05), which were present as trend (*P* = 0.01). Source data are provided as a Source data file. ADHD Attention-Deficit/Hyperactivity Disorder, ASD Autism Spectrum Disorder, EA educational attainment, MVR multivariable regression; *P*_thr_ *P*-value threshold, SNP single-nucleotide polymorphism, woADHD without ADHD.

Adopting a complementary strategy to identify variants contributing to the discordant polygenic overlap with EA, we applied a conditional *P*-value thresholding approach. Here, we systematically assessed the overlap between ASD (G_i_) and ADHD (G_j_) variant sets, based on ASD(iPSYCH,woADHD) and ADHD(iPSYCH) summary statistics, by creating a grid of marker subsets (Supplementary Fig. 1g, h). Starting with ASD-related alleles (G_i_: *P*_thr_ < 0.0015, N_SNPs_ ≤ 1,973), we, conditionally, filtered for joint association with ADHD risk at six different thresholds (0.0015 ≤ *P*_thr_ < 0.5), resulting in six G_i|j_ subsets (Fig. 4a). These sets captured 4.2–46.3% of the discovery G_i_ markers. Vice versa, we created six conditional ADHD variant subsets (G_j|i_), based on joint association with ASD risk, representing 3.1–37.5% of the discovery ADHD variant set G_j_ (*P*_thr_ < 0.0015, N_SNPs_ ≤ 2,717, Fig. 4b). As all conditional variant sets are nested within each other, we applied the multiple-testing-threshold for discovery analyses (0.0023).

Fitting a series of MVRs with conditional variant subsets G_i|j_ and G_j|i_, increased the size of estimated polygenetic association effects with EA up to five times compared to the polygenic effects observed in discovery analyses, with non-overlapping 95% confidence intervals (Fig. 4c, Supplementary Tables 10–11). The largest association effects with EA were identified with the most stringently defined G_i|j_ and G_j|i_ sets, meeting a joint selection threshold of *P*_thr_ < 0.0015 for both, ASD and ADHD risk. For example, using ASD-MVR with conditional subsets G_i|j_ (N_SNPs_ = 83, *P*_thr_ < 0.0015 for ASD and ADHD risk), we estimated ASD effects of 0.15(SE = 0.025)(*P* = 1 × 10^−7^) and ADHD effects of −0.15(SE = 0.025) (*P* = 1 × 10^−7^) years of schooling, per log odds in ASD and ADHD liability, respectively (Fig. 4c, Supplementary Table 10). Findings for ADHD-MVR with G_j|i_ were highly similar in size and strength (Fig. 4c, Supplementary Table 11).

**Fig. 5:**
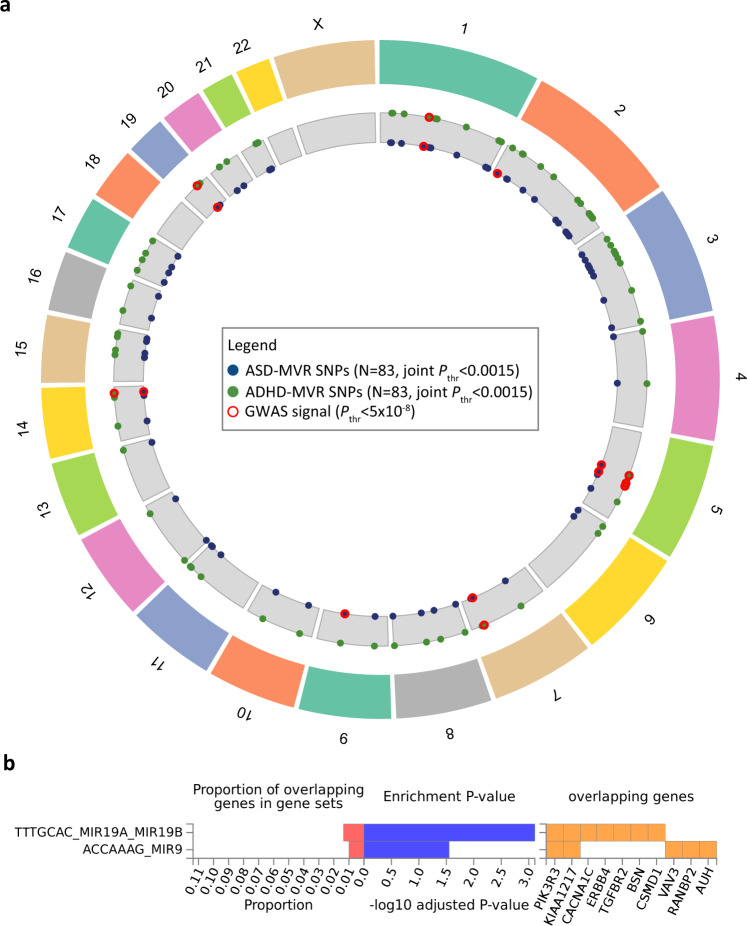
Characterisation of loci (N = 83) contributing to polygenic pleiotropy. (**a**) Chromosomal position and (**b**) functional enrichment for micro-RNA targets of variants selected at a joint *P*-value threshold for both ASD and ADHD (*P*_thr_ < 0.0015). For enrichment analyses variants were mapped to 52 genes, and, of those, 45 were aligned to unique Ensembl IDs (v92) and subjected to gene-set enrichment analysis (requesting at least 5 overlapping genes) within the Molecular Signature Database (v7.0), WikiPathways (v20191010) and reported genes from the GWAS Catalog (e96_2019-09-24) using FUMA software (v1.3.6a). Evidence for enrichment was assessed using competitive gene-set analysis as implemented in MAGMA (v1.08) using a one-sided hypergeometric test. The false discovery rate (FDR) was controlled using the Benjamini–Hochberg procedure (FUMA, v1.3.6a). The strongest evidence for enrichment was found for micro RNA target TTTGCAC_MIR19A_MIR19B (Enrichment TTTGCAC_MIR19A_MIR19B *P*_FDR-adjusted_ = 7.7 × 10^−4^, out of 515 genes). Among other categories, enrichment was also found for ACCAAAG_MIR9 (Enrichment ACCAAAG_MIR9 *P*_FDR-adjusted_ = 0.028, out of 500 genes). Source data are provided as a Source data file. ADHD Attention-Deficit/Hyperactivity Disorder, ASD, Autism Spectrum Disorder, GWAS genome-wide association study, MVR multivariable regression, *P*_thr_ *P*-value threshold; SNP, single-nucleotide polymorphism.

At the joint selection threshold of *P*_thr_ < 0.0015 for both, ASD and ADHD risk, G_i|j_ (4.2% of G_i_) and G_j|i_ (3.1% of G_j_) comprise the same 83 loci, based on 30 identical and 53 tagged proxy SNPs (LD-r^2^ = 0.6, 500 kb window), of which 99% carry the same risk-increasing allele for both disorders (Supplementary Data 1). The 83 loci were spread across the genome (Fig. 5a), including SNPs residing within the three high-confidence gwas-pw LD regions, and mapped to at least 52 genes based on position (RefSeq genes, Build37, Supplementary Data 2). This combination of 83 marker alleles is unlikely to arise due to chance, as shown by permutations (Supplementary Table 12, empirical *P* < 2 × 10^−4^), and suggests locus specificity. Nine of 83 loci represent previously reported GWAS signals (Fig. 5a), including the strongest single-variant gwas-pw signal rs4916723 (see above). This SNP is associated with ADHD risk^21^, neuroticism^38^ and alcohol consumption^39^, and has been recently identified as a pleiotropic locus contributing risk to multiple psychiatric conditions^4^ (Supplementary Data 2). rs4916723 locates within an intronic region of the *LINC00461* gene, the promoter region of the *pri-miR-34b/c* gene and about 108 kb downstream of the *MIR9-2* gene. Furthermore, using FUMA^40^ software, we found the strongest enrichment for microRNA targets when screening the Molecular Signature Database (v7.0), WikiPathways (v20191010) and reported genes from the GWAS Catalog (e96_2019-09-24), rendering patterns of spurious pleiotropy of unconnected genes less likely and strengthening support for the regulatory role of micro RNAs in disorder and behaviour. The most strongly enriched genetic feature includes MIR19A/19B targets (false discovery rate (FDR)-adjusted *P*-value = 7.7 × 10^−4^) at genes such as *CACNAC1* and *ERBB4* (Fig. 5b). In addition, we found enrichment for MIR9 targets (FDR-adjusted *P*-value = 0.028)(Fig. 5b) and 16 further categories, including for example genes related to intelligence (FDR-adjusted *P*-value = 0.0048) (Supplementary Table 13).

### Specificity analyses

Next, we investigated whether MVR findings for EA (dependent variable) extend to general intelligence, using summary statistics from intelligence(CTG) instead of EA (Supplementary Fig. 1i–j, Supplementary Table 14). These analyses confirmed discordant association patterns for both ASD and ADHD risk (multiple-testing-adjusted significance threshold: 0.0125 (0.05/4), Supplementary Table 15), suggesting generalisability of our findings to cognitive functioning.

Finally, we assessed whether EA-related variation across ASD and ADHD genetic architectures is shared with adult-onset psychiatric conditions, such as MDD, schizophrenia or BD (Supplementary Fig. 1k–l, Supplementary Table 14, multiple-testing-adjusted significance threshold: 0.0042 (0.05/12)). These exploratory analyses showed that several adult-onset disorders share EA-related variation with either an ASD or ADHD genetic architecture, or both, including discordant association patterns (Supplementary Fig. 5, Supplementary Tables 16–17, Supplementary Note 2). Meta-analysing summary statistics for disorder pairs with discordant EA-related polygenic effects, while allowing for sample overlap, accurately predicted attenuation of genetic correlations with EA (Supplementary Fig. 6).

MVR effects estimated within specificity analyses agreed with corresponding LDSC genetic correlations (Supplementary Tables 5–6, 18).

## Discussion

Using a multivariate analysis approach, we investigated genetic mechanisms embedded in ASD and ADHD genetic architectures that present as discordant polygenic association pattern with EA. We found strong evidence that EA-related genetic variation is shared across ASD and ADHD architectures, consistent with biological pleiotropy or high-LD co-localisation of genetic effects at the same subthreshold ASD- or ADHD-risk associated marker alleles. Discordant EA-related association patterns for ASD and ADHD genetic effects were (i) reciprocally detectable with MVR using either ASD- or ADHD-related risk alleles as selected for polygenic scoring approaches^34^ (ii) replicated at *P*_thr_ < 0.05 using ASD(PGC) summary statistics, (iii) consistent with the previously reported genetic overlap between EA, ASD and ADHD^17,21^, and (iv) independent of the harmonisation of GWAS marker alleles according to ASD or ADHD risk.

Discordant polygenic association with EA, across the same variants, was detected for both ASD and ADHD risk using independent samples, with little evidence for variance inflation or attenuation of signal in multivariate analyses. The observed polygenic effects are, thus, fully independent, irrespective of positive or zero genetic correlations between ASD and ADHD. The discordant polygenic association patterns remained robustly detectable when markers with alleles conferring opposite directional ASD and ADHD effects were excluded. At the single-variant level, these findings are either consistent with identical ASD and ADHD risk alleles that reflect biological pleiotropy, including special cases of GxE^41^ (scenario V, Fig. 1i), or they suggest independent causal ASD and ADHD alleles that reside in close proximity within the same gene (high-LD co-localisation, scenario IV, Fig. 1g), adhering here to a wider gene-based definition of biological pleiotropy. ASD and ADHD risk alleles might even be fully independent of each other, despite correlations across single marker effects, due to patterns of high LD^42^ between each risk allele and the assessed marker. Thus, while the same GWAS marker alleles each tag an infinitesimally small increase in risk for both disorders, they also contribute to aggregate association patterns that, jointly, differ in their polygenic nature, suggesting an overarching polygenic form of pleiotropy.

Consequently, once ASD and ADHD summary statistics are meta-analysed, discordant polygenic association effects across EA-related regions may result in a cancellation of signal and attenuate the genetic overlap with EA, as observed in this work. This implies that polygenic pleiotropy across EA-related genomic regions may affect the detectable genomic overlap between ASD and ADHD architectures, consistent with reports of positive genetic correlation between ASD and ADHD only, once latent genetic socio-economic status variance has been partialled out^43^. Our exploratory analyses furthermore suggest that EA-related polygenic pleiotropy might contribute more widely to the genetic architecture of several adult-onset disorders.

Against the shared polygenic background involving several thousands of subthreshold ASD- and/or ADHD-risk associated variants, a small fraction (<5%, N_SNPs_ = 83) passed a joint ASD and ADHD risk variant selection threshold (*P*_thr_ < 0.0015) and captured considerably larger polygenic effects than observed in discovery analyses. This set of loci encompasses variants from genomic regions on chromosome 1, 5 and 20 that were identified with high posterior probability for pleiotropy or high-LD co-localisation using gwas-pw^36^ and harbours known GWAS signals for ASD^17^, ADHD^21^ and EA^44^. Biological annotation of the 83 loci suggested a role for microRNA genes. One of these loci is rs4916723, the single variant with the highest posterior probability for pleiotropy/high-LD co-localization as identified by gwas-pw, which resides near/within *LINC00461*, *pri-miR-34b/c* and *MIR9-2* genes. miR-9 couples brain neurogenesis and angiogenesis in vertebrates, and is involved in a conserved transcriptional cascade that is critical for brain development^45^. Consistently, mapping the full set of 83 loci to at least 52 genes, the strongest enrichment was found for MIR19A/19B target genes. miRNAs are key regulators of many biological processes in neurodevelopment^46^ and involve post-transcriptional regulation of gene expression (often through gene silencing), while their expression can also be shaped by environmental signals^46^. The identified enriched miRNA targets encode, for example, biological signalling proteins such as the calcium voltage-gated channel subunit alpha1C (CACNA1C) and the tyrosinkinase ERBB4, which have been previously associated with both ASD and ADHD as well as other disorders^35,47,48^.

Ultimately, an enrichment in miRNA targets is consistent with multiple regulatory sites in close genomic proximity (scenario IV, Fig. 1g) or different regulations of the same site (scenario V, Fig. 1i), but not with spurious pleiotropy due to functionally unrelated causal genetic variants in high LD (scenario IV, Fig. 1h). Thus, our results provide support for a recently proposed class of genetic influences for psychiatric illness which does not confer broad liability to disorder but is thought to shape the phenotype expression through direct and interactive genetic effects or environmental factors^4^. As construed by the omnigenic model^49^, such ‘peripheral’ genetic influences, acting through trans effects, could control shared ADHD/ASD ‘core’ variation.

Adopting a statistical framework developed for Mendelian Randomization analyses^32^, this study disentangled ASD and ADHD effects at the same GWAS marker allele and identified independent polygenic associations with EA for ASD and ADHD risk. Evidence for discordant EA-related associations for ASD and ADHD encoded at the same GWAS marker alleles was replicated using two independent ASD collections (variant selection threshold *P*_thr_ < 0.05). This suggests that our findings are robust and unlikely to be affected by diagnostic classification systems for clinical ASD, routes of patient ascertainment or association analysis designs. Moreover, we show here that patterns of polygenic pleiotropy become detectable once SNP estimates from multiple disorders are modelled simultaneously, using multivariate approaches that can detect and de-stratify polygenic signals. For example, across iPSYCH samples discordant ASD and ADHD association effects with EA increased in strength and size once modelled in a multivariate compared to a univariate regression framework (Supplementary Tables 3–4) indicating here, potentially, negative confounding effects. The possibility that our findings are affected by ascertainment bias (scenario II, Fig. 1c) is not very likely, as we observed little evidence for negative covariance or attenuation of signal when ASD and ADHD risk effects were modelled simultaneously. Furthermore, ASD and ADHD symptom heterogeneity may shape the genetic overlap between neurodevelopmental disorders, EA and cognition-related traits^17,50^ and future studies with access to this information are warranted to fully understand the underlying complexity of multivariate inter-correlations. Finally, ADHD-MVR, but not ASD-MVR analyses, may suffer from winner’s curse^51^ due to a lack of an independent ADHD sample. In addition, the candidacy of miRNAs functionality underlying polygenic pleiotropy requires replication in larger ASD and ADHD cohorts that are currently not yet publicly available.

Our findings show that EA-related polygenic variation is shared across ASD and ADHD genetic architectures and that combinations of the same risk alleles, through mechanisms consistent with biological pleiotropy or high-LD co-localisation at the single-variant level, can encode ASD-related positive and ADHD-related negative associations with EA, without involving further loci. These independent aggregate effects across shared EA-related marker alleles suggest a polygenic form of pleiotropy that shapes the detectable genome-wide genetic overlap between ASD and ADHD and is consistent with effect cancellation across EA-related regions.

## Methods

### Data sets

Genome-wide SNP information on EA, intelligence and neuropsychiatric disorders was obtained from GWAS summary statistics^17,20,21,35,44,52,53^ (Table 1, Supplementary Table 14).

#### EA and intelligence

GWAS summary statistics on years of schooling (excluding 23andMe) were obtained from the Social Science Genetic Association Consortium (SSGAC, https://www.thessgac.org/, Table 1)^44^. EA was coded according to the International Standard Classification of Education (1997) scale^44^ and analysed as a quantitative variable defined as an individual’s years of schooling. Participants were >30 years of age at the time of assessment and of European ancestry. The meta-analysis consisted primarily of population-based cohorts, but also included family-based and case-control samples. 55.2% of participants were female. For most cohorts, genome-wide data were imputed to a 1000 genomes project version 3 reference template^44^.

GWAS summary statistics on intelligence^20^ were retrieved from the Complex Trait Genetics (CTG) lab (https://ctg.cncr.nl/software/summary_statistics, Supplementary Table 14). Participating cohorts were primarily population-based. Each cohort assessed intelligence with different instruments that were re-defined to index a common latent factor of general intelligence^20^. Participants had a wide age range (from 5 to 98 years), 51.2% were female and all of them were of European descent. Genome-wide data were predominantly imputed to the Haplotype Reference Consortium (HRC) reference panel^20^.

#### ASD and ADHD

GWAS summary statistics for ASD and ADHD were accessed through the Danish Lundbeck Foundation Initiative for Integrative Psychiatric Research (iPSYCH, http://ipsych.au.dk/) using samples from the Danish Neonatal Screening Biobank hosted by Statens Serum Institute^17,21,54^ (ASD(iPSYCH, woADHD), ADHD(iPSYCH), Table 1). iPSYCH adopts a case-control design (26.6% female ASD-cases^17^, 21.6% female ADHD-cases^21^) with shared controls (~49% female)^17,21^, all of European ancestry with age ranges spanning infancy to adulthood^17,21^. ASD samples were restricted to ASD-cases without (wo) an additional ADHD diagnosis (ASD(iPSYCH,woADHD), Table 1) to avoid overlap with ADHD(iPSYCH). However, ADHD-cases may have an additional ASD diagnosis. Information on ADHD cases without ASD was not available.

ASD cases and ADHD cases were diagnosed according to ICD-10^55^ and identified using the Danish Psychiatric Central Research Register^56^. Registry-based ASD diagnoses were validated previously^17,21^. Controls were randomly selected from the same nationwide birth cohort and did not have a diagnosis of ASD or ADHD or moderate-severe mental retardation (F71-F79)^17,21,54^. The median age at first diagnosis of ASD was 10 years. Genotyping was performed using the Illumina Infinium PsychArray BeadChip and genotypes were imputed to a 1000 Genomes template (Phase3, release 02-05-2013). Genotyping, quality control, imputation and genetic association analysis were carried out using the Ricopili pipeline with standard PGC settings^17,21^.

Independent ASD GWAS summary statistics were obtained from the Psychiatric Genomics Consortium (PGC, www.med.unc.edu/pgc/). They were based on a case-control/pseudo-control design and all individuals were ≥3 years of age and of European ancestry (ASD(PGC), Table 1). Information on the male-female ratio was not available^35^. A consensus ASD diagnosis was made using research standard diagnoses and expert clinical consensus diagnoses. The majority of ASD-cases (94.1%) also had a clinical diagnosis based on the Autism Diagnostic Interview-Revised^57^ or the Autism Diagnostic Observation Schedule^58^. Genome-wide data were imputed to a 1000 Genomes reference template (Phase1 v3). Note that the sample size for ADHD(iPSYCH) is about three times compared to ASD(PGC).

#### Sample overlap

GWAS summary statistics for ASD(PGC)^35^, ASD(iPSYCH, woADHD)^17^ and ADHD(iPSYCH)^21^ (Table 1) were independent from EA^44^ and intelligence^20^. ASD(PGC), ADHD(iPSYCH) and ASD(iPSYCH,woADHD)^17^ GWAS statistics have independent case samples; iPSYCH controls were shared across reported ASD(iPSYCH,woADHD) and ADHD(iPSYCH) summary statistics.

#### Specificity analyses including other psychiatric disorders

To assess the specificity of MVR association profiles, we also investigated GWAS summary statistics for MDD^52^, schizophrenia (SCZ)^53^ and BD^53^. Cases were identified based on international consensus criteria. For MDD, cases were identified based on a lifetime diagnosis of MDD, established using DSM-III, DSM-IV, ICD-9 and/or ICD-10 criteria or self-report^52^. For SCZ, the majority of cases were diagnosed using DSM-III, DSM-III-R, DSM-IV, ICD-10, and SCID criteria^53,59^. BD cases were diagnosed according to DSM-III, DSM-IV-TR, DSM-IV, SCID, ICD-10 or RDC criteria^53,60^. For all three data sets, genotype imputation was performed using the IMPUTE2/SHAPEIT pipeline against the 1000 Genomes Project (v3) template. Summary data were obtained from the PGC (www.med.unc.edu/pgc/, Supplementary Table 14), all based on participants of European ancestry. For these analyses, the sample overlap is more pronounced, as some cohorts, such as UKBiobank, are shared across EA, MDD, SCZ or BD summary statistics (but not ASD and ADHD). Presented MVR studies for adult-onset disorders have thus an exploratory character only. However, the case overlap of ASD(iPSYCH,woADHD) and ADHD(iPSYCH) with MDD, SCZ or BD cases is at most 2%, similar to recent cross-disorder analyses^4^ (Table 1, Supplementary Table 14). Likewise, iPSYCH controls were shared across reported ASD(iPSYCH,woADHD), ADHD(iPSYCH), MDD, SCZ and BD summary statistics, as in recent cross-disorder analyses^4^.

#### Cross-disorder meta-analyses

For genetic correlation analyses with EA, cross-disorder GWAS summary statistics were derived for pairs of disorders that reveal opposite genetic association patterns with EA across shared marker alleles, as predicted with MVR. This included four effect-size based meta-analyses, allowing for sample overlap: (i) ASD(woADHD, iPSYCH) and ADHD(iPSYCH); (ii) ASD(PGC) and ADHD(iPSYCH); for specificity analyses only, we also analysed (iii) ASD(woADHD, iPSYCH) and MDD (PGC); and (iv) ADHD(iPSYCH) and BD(PGC), as integrated within METACARPA software^61^.

### SNP-heritability and genetic correlations

SNP-h^2^, the proportion of phenotypic or liability variance tagged by SNPs on genotyping arrays, was estimated for EA, intelligence and psychiatric disorders using linkage disequilibrium score (LDSC) regression^62^ (Supplementary Table 19). To estimate SNP-h^2^, genome-wide *χ*^2^-statistics are regressed on the amount of genetic variation captured by each SNP^62^, while the intercept of this regression minus one is an estimator of the mean contribution of confounding bias to the inflation in the mean *χ*^2^-statistic^62^. SNP-h^2^ was calculated on the liability scale for psychiatric disorder samples, assuming a population prevalence of 0.012 for ASD^17^, 0.05 for ADHD^63^, 0.162 for MDD^64^, 0.007 for SCZ^65^ and 0.006 for BD^66^.

In extension, unconstrained LDSC correlation^67^ analysis was applied to estimate bivariate genetic correlations (*r*_g_) among psychiatric disorders, between psychiatric disorders (including cross-disorder meta-analyses) and EA, as well as between psychiatric disorders and intelligence (Supplementary Tables 5–6, 18). This involves a regression of the product of test statistics on LD score and captures the extent of shared genetic influences between phenotypes assessed in different samples^67^. The multiple-testing-adjusted significance threshold was determined at 0.002, correcting for a total of 25 LDSC correlation analyses in this work.

All analyses were performed with LDSC software^62,67^ (v1.0.0) and based on the set of well-imputed HapMap3 SNPs and a European reference panel of LD scores^67^.

### Multivariable regression analyses

We adopted a bidirectional inverse-variance weighted regression framework, MVR, analogous to statistical models proposed for multivariable MR^32^. This approach was implemented using GWAS summary statistics, often described as Egger regression^32^. Here, MVR analyses do not infer causality as we allow for biological pleiotropy. We apply this method to simultaneously estimate genetic ASD and ADHD risk associations with EA. We control for collider bias that may arise when adjusting for heritable covariates^33^ by studying relationships between genetically predicted phenotypes only.

#### Discovery analyses (Supplementary Fig. 1a, b)

As a first step, we selected ASD-related (G_i_) and ADHD-related (G_j_) variant sets, according to guidelines for polygenic scoring methods^34^. ASD-related variant sets G_i_ (with i = 1, …, m SNPs) and ADHD-related variant sets G_j_ (with j = 1, …, n SNPs) were selected from ASD(iPSYCH,woADHD) and ADHD(iPSYCH) GWAS statistics respectively, using 11 different *P*-value thresholds (*P*_thr_, 5 × 10^−8^; 5 × 10^−7^; 5 × 10^−6^; 5 × 10^−5^; 0.0005; 0.0015; 0.005; 0.05; 0.1; 0.3; 0.5). All variant sets were restricted to common (minor allele frequency>0.01), independent (LD-r^2^ < 0.25 within ±500 kb) and well-imputed (imputation quality(INFO) > 0.7) SNPs. Next, corresponding SNP estimates for ASD (${\hat{\beta }}_{{{{{{\rm{ASD}}}}}}}$_,_) ADHD (${\hat{\beta }}_{{{{{{\rm{ADHD}}}}}}}$) and EA (${\hat{\beta }}_{{{{{{\rm{EA}}}}}}}$) were extracted from ASD(iPSYCH,woADHD), ADHD(iPSYCH) and EA(SSGAC) GWAS statistics. Then, we fitted an ASD-MVR for each ASD variant set G_i_, as follows:

1 $${\hat{\beta }}_{{{{{{{\rm{EA}}}}}}}_{i}}={\theta }_{0\ast }+{\theta }_{{{{{{\rm{ASD}}}}}}}{\hat{\beta }}_{{{{{{{\rm{ASD}}}}}}}_{i}}+{\theta }_{\ast {{{{{\rm{ADHD}}}}}}}{\hat{\beta }}_{{{{{{{\rm{ADHD}}}}}}}_{i}}$$

2 $${{{{{\rm{weig}}}}}}{{{{{\rm{h}}}}}}{{{{{\rm{ts}}}}}}={{{{{\rm{se}}}}}}{({\hat{\beta }}_{{{{{{{\rm{EA}}}}}}}_{i}})}^{-2}$$

where ${\hat{\beta }}_{{{{{{{\rm{EA}}}}}}}_{i}}$ (dependent variable) are SNP estimates for EA, ${\hat{\beta }}_{{{{{{{\rm{ASD}}}}}}}_{i}}$ (independent variable) are SNP estimates for ASD and ${\hat{\beta }}_{{{{{{{\rm{ADHD}}}}}}}_{i}}$ (independent variable) are SNP estimates for ADHD. Within this MVR framework, based on ASD variant set G_i_, ${\theta }_{0\ast }$ is the regression intercept, ${\theta }_{{{{{{\rm{ASD}}}}}}}$ the ASD effect and ${\theta }_{\ast {{{{{\rm{ADHD}}}}}}}$ the ADHD effect, weighted by the inverse variance of the dependent variable, consistent with the statistical framework of Egger regression based MVR analyses^32^. The intercept ${\theta }_{0\ast }$ is an estimate of *α*_*i*_’^32^, the direct pleiotropic influences between the analysed variants G_i_ and EA that are neither captured by ${\theta }_{{{{{{\rm{ASD}}}}}}}$ nor ${\theta }_{\ast {{{{{\rm{ADHD}}}}}}}$.

Similarly, for each ADHD variant set G_j_, an ADHD-MVR was fitted as follows:

3 $${\hat{\beta }}_{{{{{{{\rm{EA}}}}}}}_{j}}={\theta }_{0{{{{{\rm{\#}}}}}}}+{\theta }_{{{{{{\rm{ADHD}}}}}}}{\hat{\beta }}_{{{{{{{\rm{ADHD}}}}}}}_{j}}+{\theta }_{{{{{{\rm{\#}}}}}}{{{{{\rm{ASD}}}}}}}{\hat{\beta }}_{{{{{{{\rm{ASD}}}}}}}_{j}}$$

4 $${{{{{\rm{weights}}}}}}={{{{{\rm{se}}}}}}{({\hat{\beta }}_{{{{{{{\rm{EA}}}}}}}_{j}})}^{-2}$$

where ${\hat{\beta }}_{{{{{{{\rm{EA}}}}}}}_{j}}$ (dependent variable) are SNP estimates for EA, ${\hat{\beta }}_{{{{{{{\rm{ADHD}}}}}}}_{j}}$ (independent variable) are SNP estimates for ADHD and ${\hat{\beta }}_{{{{{{{\rm{ASD}}}}}}}_{j}}$ (independent variable) are the SNP estimates for ASD. For this MVR framework, using ADHD variant set G_j_, ${\theta }_{0\#}$ is the regression intercept, ${\theta }_{{{{{{\rm{ADHD}}}}}}}$ the ADHD effect and ${\theta }_{{{{{{\rm{\#}}}}}}{{{{{\rm{ASD}}}}}}}$ the ASD effect. The intercept ${\theta }_{0\#}$ is an estimate of *α*_j_’^32^, the direct pleiotropic influences between the analysed variants G_j_ and EA that are neither captured by ${\theta }_{{{{{{\rm{ADHD}}}}}}}$ nor ${\theta }_{{{{{{\rm{\#}}}}}}{{{{{\rm{ASD}}}}}}}$

Reported MVR effects ($\hat{\theta }$) present changes in years of schooling, either per increase in log odds ASD or ADHD liability, pooled across the variant set. The overall MVR model fit was compared to univariable models (see below) using likelihood-ratio tests, as implemented in the R:stats library (Rv3.5.1). Collinearity between independent variables was assessed by the VIF(R:car library (Rv3.5.1)).

Multivariable MR Egger-related approaches with intercept terms, including MVR analyses applied in this study, are sensitive to allelic alignment. It has been recommended to orient all variants with respect to the genetic association with the independent variable of primary interest^32^. Thus, SNP estimates were aligned to increase ASD risk in ASD-MVRs and ADHD risk in ADHD-MVRs. For simplicity, MVR findings in the main manuscript are presented for two *P*-value thresholds only: *P*_thr_ < 0.0015, consistent with conservative selection thresholds recommended for polygenic scoring approaches^34^, and *P*_thr_ < 0.05, a less stringent threshold that has been previously selected to study polygenic scores in complex psychiatric disorders^59^, to increase the statistical power and precision of MVR estimates. In total, 22 tests were performed as part of discovery analyses (two MVR models across 11 variant sets), resulting in a multiple-testing-adjusted significance threshold of 0.0023.

To assess whether EA-association patterns with ASD and ADHD were driven by variants that encoded opposite risk alleles for each disorder (scenario III, Fig. 1d–f), discovery MVR analyses were repeated with concordant variant sets. Concordant variant sets (*P*_thr_ < 0.0015 and *P*_thr_ < 0.05) were created by restricting ASD variant sets G_i_ and ADHD variant sets G_j_ to variants with the same risk-increasing allele for both ASD and ADHD risk. Consequently, by design, SNP estimates were aligned to increase both ASD and ADHD risk in these analyses. As all variant sets were nested within the variant sets used for discovery analysis, the same multiple-testing-adjusted significance threshold was applied (0.0023).

#### Follow-up analyses with ASD(PGC) (Supplementary Fig. 1c, d)

To replicate MVR findings, ASD-MVR and ADHD-MVR were conducted using ASD SNP estimates ($\hat{\beta }$_ASD_) from ASD(PGC), instead of ASD(iPSYCH,woADHD), for ASD and ADHD variant sets from the discovery analyses selected at *P*_thr_ < 0.0015 and *P*_thr_ < 0.05. Within ASD-MVR, SNP estimates were aligned to increase ASD risk as observed in ASD(PGC). In total, four tests were performed (two MVR models across two variant sets), resulting in a multiple-testing-adjusted significance threshold of 0.0125.

As for discovery analyses, concordant variant sets were created by restricting ASD (G_i_) and ADHD (G_j_) variant sets to variants with the same risk-increasing allele for ASD and ADHD risk (multiple-testing-adjusted significance threshold 0.0125).

#### ASD-MVR and ADHD-MVR analyses using variants from LD blocks with high posterior probability for pleiotropy or high-LD co-localisation (Supplementary Fig. 1e, f)

Gwas-pw^36^ (v0.21) analyses were based on summary statistics for (i) ASD(iPSYCH,woADHD) and ADHD(iPSYCH), and (ii) ASD(PGC) and ADHD(iPSYCH), without applying *P*-value selection criteria. After dividing the genome into approximately independent LD blocks, gwas-pw estimates the posterior probability that a given LD block contains: (model 1) a genetic variant associated with ASD, (model 2) a genetic variant associated with ADHD, (model 3) a genetic variant associated with both disorders, reflecting biological pleiotropy (scenario V, Fig. 1i) or high-LD co-localisation (scenario IV Fig. 1g, h), or (model 4) multiple genetic variants that are each associated with a different disorder (co-localisation in the presence of low/moderate LD). Evidence for model 3 and model 4 was evaluated at a stringent posterior probability threshold (>0.9), and correction for overlapping samples between summary statistics was applied based on LDSC genetic correlation estimates (Supplementary Table 5), consistent with author recommendations^36^. From the identified LD blocks, we extracted independent ASD- and ADHD-related variants (LD-r^2^ < 0.25 within ±500 kb), and carried out ASD-MVR and ADHD-MVR as described for discovery analyses above (Supplementary Fig. 1e–f). To enhance stringency, we applied the multiple-testing-adjusted significance threshold of 0.0023, as derived for discovery analyses.

#### Identification of single variants using conditional *P*-value thresholding (Supplementary Fig. 1g, h)

To identify variants underlying the discordant EA-related association patterns for ASD and ADHD genetic risk, we systematically assessed overlap between ASD (G_i_) and ADHD (G_j_) variant sets. Overlapping independent SNPs associated with both ASD and ADHD risk were identified using PLINK (v1.90b3w, https://www.cog-genomics.org/plink2; 500 kb and LD-r^2^ ≥ 0.6), starting with ASD and ADHD variant sets from discovery analyses at *P*_thr_ < 0.0015. For each variant set (ASD (G_i_, N_SNPs_ = 1,973) and ADHD (G_j_, N_SNPs_ = 2,717)), we identified SNPs that were, conditionally, also associated with the other disorder across six different *P*-value thresholds (0.0015; 0.005; 0.05; 0.1; 0.3; 0.5). This resulted in six subsets for ASD-related variants (G_i|j_) and six subsets for ADHD-related variants (G_j|i_). As conditionally selected variant sets are nested within each discovery set G_i_ and G_j_, respectively, the multiple-testing-adjusted threshold for discovery analyses was applied (0.0023). Note that the notation of conditional variant sets has no mathematical meaning.

To assess whether association with EA across the set of 83 identified loci (G_i|j_, G_j|i_, ASD and ADHD *P*_thr_ < 0.0015, N_SNPs_ = 83) may occur by chance, we performed a permutation analysis. For each permutation, 83 independent SNPs were randomly selected from G_i_ (*P*_thr_ < 0.0015, ASD(iPSYCH, woADHD)) for ASD-MVR, and, likewise, from G_j_ (*P*_thr_ < 0.0015, ADHD(iPSYCH) GWAS) for ADHD-MVR. Corresponding SNP estimates for ASD ($\hat{\beta }$_ASD_), ADHD ($\hat{\beta }$_ADHD_) and EA ($\hat{\beta }$_EA_) were extracted from ASD(iPSYCH, woADHD), ADHD(iPSYCH) and EA(SSGAC) GWAS statistics, respectively. In total, 10,000 permutations were carried out for both, empirical ASD-MVR and ADHD-MVR analyses.

#### Specificity analyses related to general intelligence (Supplementary Fig. 1i, j)

ASD-MVR (G_i_ discovery set; *P*_thr_ < 0.0015 and *P*_thr_ < 0.05) and ADHD-MVR models (G_j_, discovery set; *P*_thr_ < 0.0015 and *P*_thr_ < 0.05) were fitted to genetically predictable general intelligence as outcome ($\hat{\beta }$_Intelligence_) instead of EA ($\hat{\beta }$_EA_), using Intelligence(CTG) GWAS summary statistics. For these four additional tests (two MVR models across two variant sets), we applied a multiple-testing-adjusted significance threshold of 0.0125.

#### Specificity analyses related to risk for other psychiatric disorders (Supplementary Fig. 1k, l)

To assess whether ASD- (G_i_) and ADHD-related variant sets (G_j_), encode also discordant EA-related association patterns for adult-onset neuropsychiatric disorders (MDD, SCZ or BD), we carried out sensitivity analyses. For risk-related alleles within the discovery sets G_i_ and G_j_ (at *P*_thr_ < 0.0015 and *P*_thr_ < 0.05), we extracted SNP estimates for MDD ($\hat{\beta }$_MDD_), SCZ ($\hat{\beta }$_SCZ_) or BD ($\hat{\beta }$_BD_) from GWAS summary statistics for MDD(PGC), SCZ(PGC), BD(PGC), respectively. Using ASD-MVR, polygenic ASD effects with EA (*θ*_ASD_) were estimated simultaneously with the polygenic effect of another psychiatric disorder (θ_*MDD_, θ_*SCZ_ or θ_*BD_). Likewise, using ADHD MVR (*θ*_ADHD_), polygenic ADHD effects on EA were simultaneously estimated with risk effects for other psychiatric disorders (*θ*_#MDD_, *θ*_#SCZ_ or *θ*_#BD_). In total, 12 sensitivity analyses were performed, leading to a multiple-testing-adjusted *P*-value threshold of 0.0042.

### Univariable regression models

To assess the robustness of MVR findings, weighted univariable models were included in discovery, follow-up and specificity analyses (see above, Supplementary Fig. 1a–f, i–l). Univariable regressions include the same dependent variable as MVR (EA or general intelligence), but estimate only one polygenic disorder-related effect. Univariable and MVR model fit was compared using a likelihood-ratio test as implemented in the R:stats library (Rv3.5.1).

### SNP annotations

The 83 loci identified using a conditional thresholding approach (see above, ASD *P*_thr_ < 0.0015 and ADHD *P*_thr_ < 0.0015) were annotated (Supplementary Data 2). Frequencies for the modelled allele were extracted from the Haplotype Reference Consortium r1.1^68^ and GWAS hits (*P* < 5 × 10^−8^), as recorded in the GWAS Catalog, were identified using the UCSC Genome Browser data integrator tool, build 37 (https://genome.ucsc.edu/cgi-bin/hgIntegrator). Finally, SNPs were mapped to 52 RefSeq gene IDs (genome build 37) based on positional mapping using PLINK software (v1.90b3w, https://www.cog-genomics.org/plink2, 0 kb gene window), similar to the default options in MAGMA gene-enrichment software^69^.

### Gene-set enrichment analyses

Gene-set enrichment (>5 overlapping genes, FDR-adjusted *P*-values) was conducted with respect to pre-defined gene-sets derived from the Molecular Signature Database (v7.0), WikiPathways (v.20191010), or the GWAS Catalog (v.e96_r2019-09-24). Enrichment analysis was carried out with MAGMA^69^ (v1.08) using a one-sided hypergeometric test, as implemented in FUMA^40^ software (v1.3.6a, https://fuma.ctglab.nl/), by mapping identified genes for the 83 loci (see above) to unique Ensembl IDs (v92). The False Discovery Rate (FDR) was controlled using the Benjamini–Hochberg procedure (FUMA, v1.3.6a).

All used web resources are listed in Supplementary Note 3.

### Reporting summary

Further information on research design is available in the Nature Research Reporting Summary linked to this article.

## Supplementary information

Supplementary Information

Description of Additional Supplementary Files

Supplementary Data 1

Supplementary Data 2

Reporting Summary

## Data Availability

Publicly available GWAS summary statistics were retrieved for ASD(PGC): http://www.med.unc.edu/pgc/files/resultfiles/pgcasdeuro.gz, EA(SSGAC): https://www.dropbox.com/s/ho58e9jmytmpaf8/GWAS_EA_excl23andMe.txt?dl=0, Intelligence(CTG): https://ctg.cncr.nl/documents/p1651/SavageJansen_IntMeta_sumstats.zip, MDD(PGC): 10.6084/m9.figshare.14672082, SCZ(PGC) and BD(PGC): 10.6084/m9.figshare.14672019. In order to ensure that there is no conflict with ongoing projects, collaborations and iPSYCH’s data sharing policies, restrictions apply to the availability of summary statistics from the iPSYCH sample. For access to these data, researchers should prepare a short application briefly describing the proposed study. Response will typically be within 2 weeks. Access to individual level data will in addition require institutional collaboration agreement and data use agreement following GDPR. Contact the lead principal investigator A.D.B. (anders@biomed.au.dk) for access requests. The data generated in this study are provided in the Supplementary Information/Source Data file. Source data are provided with this paper.

## Source data

Source data
