# Supplementary material for: Discordant associations of educational attainment with ASD and ADHD implicate a polygenic form of pleiotropy
Source: Nat Commun. 2021 Nov 11;12:6534. doi: 10.1038/s41467-021-26755-1 (PMC8586371; doi:10.1038/s41467-021-26755-1)
Supplement: Supplementary file 1 — Supplementary Information [file 41467_2021_26755_MOESM1_ESM.pdf]

Supplementary Information

Discordant associations of educational attainment with ASD and ADHD implicate a polygenic form of pleiotropy

Supplementary Notes ... 3

Supplementary Note 1: Risk-allele concordance between GWAS summary statistics ... 3

Supplementary Note 2: MVR specificity analyses for adult-onset psychiatric disorders ... 4

Supplementary Note 3: Web resources ... 5

Supplementary Tables ... 6

Supplementary Table 1: Discovery ASD-MVR (5x10^{-8}<P_{thr}<0.5) ... 6

Supplementary Table 2: Discovery ADHD-MVR (5x10^{-8}<P_{thr}<0.5) ... 7

Supplementary Table 3: Discovery ASD-MVR and ADHD-MVR (P_{thr}<0.0015; P_{thr}<0.05), model fit ... 8

Supplementary Table 4: Discovery ASD-MVR and ADHD-MVR (P_{thr}<0.0015; P_{thr}<0.05), concordant variants ... 9

Supplementary Table 5: Genetic correlations among psychiatric disorders ... 10

Supplementary Table 6: Genetic correlations of psychiatric disorders with educational attainment 11

Supplementary Table 7: Follow-up ASD-MVR and ADHD-MVR (P_{thr}<0.0015; P_{thr}<0.05), analyses with ASD(PGC) SNP estimates (independent variable) ... 12

Supplementary Table 8: Follow-up ASD-MVR and ADHD-MVR (P_{thr}<0.0015; P_{thr}<0.05) with ASD(PGC) SNP estimates (independent variable), concordant variants ... 13

Supplementary Table 9: ASD-MVR and ADHD-MVR with variants from LD blocks with high posterior probability for pleiotropy ... 14

Supplementary Table 10: Identification of single variants using conditional *P*-value thresholding: ASD-MVR ... 15

Supplementary Table 11: Identification of single variants using conditional *P*-value thresholding: ADHD-MVR ... 16

Supplementary Table 12: Permutation analysis ... 17

Supplementary Table 13: Gene-set enrichment analysis ... 18

Supplementary Table 14: Sample description for MDD, SCZ, BD and general intelligence ... 19

Supplementary Table 15: Specificity analyses related to general intelligence (dependent variable) using ASD-MVR and ADHD-MVR (P_{thr}<0.0015; P_{thr}<0.05) ... 20

Supplementary Table 16: Specificity analyses related to risk for other psychiatric disorders (independent variable), ASD-MVR (P_{thr}<0.0015; P_{thr}<0.05) ... 21

Supplementary Table 17: Specificity analyses related to risk for other psychiatric disorders (independent variable), ADHD-MVR (P_{thr}<0.0015; P_{thr}<0.05) ... 22

Supplementary Table 18: Genetic correlations of ASD and ADHD with general intelligence ... 23

Supplementary Table 19: SNP-heritability estimates ... 24

Supplementary Figures.. 25
Supplementary Figure 1: MVR study design.. 27
Supplementary Figure 2: Bivariate analyses of ASD, ADHD and EA genetic effects using ASD and ADHD concordant variant sets.. 28
Supplementary Figure 3: Evidence for pleiotropy and co-localisation using gwas-pw..................... 29
Supplementary Figure 4: Genes annotated within pleiotropic genomic segments.......................... 30
Supplementary Figure 5: Cross-disorder associations with educational attainment for other disorders.. 31
Supplementary Figure 6: Genetic correlations with EA for cross-disorder meta-analyses............... 33
Supplementary References.. 34

Supplementary Notes

Supplementary Note 1: Risk-allele concordance between GWAS summary statistics

Discovery analyses (Supplementary Figure 1a-b).

Among the 1,973 SNPs selected at P_{thr}<0.0015 in ASD(iPSYCH, woADHD), 87% had concordant effects with ADHD(iPSYCH). Among the 35,921 SNPs selected at P_{thr} <0.05 in ASD(iPSYCH, woADHD), 80% had concordant effects with ADHD(iPSYCH). Likewise, among the 2,717 SNPs selected at threshold P_{thr}<0.0015 in ADHD(iPSYCH), 88% had concordant effects with ASD(iPSYCH, woADHD). Among the 41,334 SNPs selected at threshold P_{thr}<0.05 in ADHD(iPSYCH), 78% had concordant effects with ASD(iPSYCH, woADHD).

Follow-up analyses with ASD(PGC) (Supplementary Figure 1c-d).

Among the 1,973 SNPs selected at P_{thr}<0.0015 in ASD(iPSYCH, woADHD), 1,886 were available in ASD(PGC) and 49% had concordant effects. Among the 35,921 SNPs selected at P_{thr}<0.05 in ASD(iPSYCH, woADHD), 33,845 were available in ASD(PGC) and 52% had concordant effects. Among the 2,717 SNPs selected at threshold P_{thr}<0.0015 in ADHD(iPSYCH), 2,627 were available in ASD(PGC) and 51% had concordant effects. Among the 41,334 SNPs selected at threshold P_{thr}<0.05 in ADHD(iPSYCH), 38,656 were available in ASD(PGC) and 52% had concordant effects.

Specificity analyses related to risk for other psychiatric disorder (Supplementary Figure 1k-l).

Among the SNPs selected at P_{thr}<0.0015 in ASD(iPSYCH, woADHD), 2,644 were available in MDD(PGC) and 75% had concordant effects, 1,786 were available in SCZ(PGC) and 53% had concordant effects, and 1,859 were available in BD(PGC) and 54% had concordant effects. Among the SNPs selected at P_{thr}<0.05 in ASD(iPSYCH, woADHD), 50,904 were available in MDD(PGC) and 65% had concordant effects, 31,026 were available in SCZ(PGC) and 52% had concordant effects, and 32,367 were available in BD(PGC) and 52% had concordant effects.

Among the SNPs selected at P_{thr}<0.0015 in ADHD(iPSYCH), 2,567 were available in MDD(PGC) and 77% had concordant effects, 2,278 were available in SCZ(PGC) and 55% had concordant effects, and

2,340 were available in BD(PGC) and 52% had concordant effects. Among the SNPs selected at P_{thr}<0.05 in ADHD(iPSYCH), 38,093 were available in MDD(PGC) and 68% had concordant effects, 31,395 were available in SCZ(PGC) and 52% had concordant effects, and 32,788 were available in BD(PGC) and 51% had concordant effects.

Supplementary Note 2: MVR specificity analyses for adult-onset psychiatric disorders

As part of exploratory specificity analyses, we investigated whether EA-related variation across ASD and ADHD genetic architectures is shared with adult-onset psychiatric conditions. We applied ASD- and ADHD-MVR using SNP estimates for major depressive disorder (MDD), schizophrenia (SCZ) and bipolar disorder (BD) aligned to ASD-related (G_i) and ADHD-related (G_j) variants, respectively (multiple-testing-adjusted significance threshold: 0.0042 (0.05/12)) (Supplementary Figure 1k-l).

These analyses showed that all studied adult-onset psychiatric disorders share EA-related variation with either an ASD or ADHD genetic architecture, or both, especially at the relaxed *P*-value threshold (P_{thr}<0.05; Supplementary Figure 5, Supplementary Table 18-19). The strongest discordant association pattern with EA was detected for ASD-related risk alleles in combination with MDD risk (ASD-MVR at P_{thr}<0.05: $\hat{\theta}_{*MDD}$=-0.012, SE=0.001, $P<1\times10^{-10}$, Supplementary Table 18), and for ADHD-related risk alleles in combination with BD risk (ADHD-MVR at P_{thr}<0.05: $\hat{\theta}_{\#BD}$=0.008, SE=0.001, $P<1\times10^{-10}$, Supplementary Table 19). All MVR effects were consistent with known LDSC genetic correlations (Supplementary Table 5-6). The observed discordant association effects with EA were weaker and smaller for adult-onset disorders compared to discovery ASD- and ADHD-MVR effects, although they are likely to be inflated due to sample overlap with EA(SSGAC). Meta-analysing summary statistics for pairs of disorders with discordant EA-related polygenic effects, while allowing for sample overlap, accurately predicted the attenuation of genetic correlations with EA (Supplementary Figure 6), especially between ADHD(iPSYCH) and BD(PGC). This may suggest universal genetic mechanisms across EA-related genomic regions, where identical GWAS marker alleles capture disorder-specific subthreshold risk effects that result in independent polygenic associations with EA.

Supplementary Note 3: Web resources

CTG lab: https://ctg.cncr.nl/software/summary_statistics

FUMA: https://fuma.ctglab.nl/

Gwas-pw: https://github.com/joepickrell/gwas-pw

iPSYCH: https://ipsych.au.dk/downloads/

LDSC: https://github.com/bulik/ldsc

PLINK: https://www.cog-genomics.org/plink2

PGC: https://www.med.unc.edu/pgc/

R: https://www.r-project.org/

SSGAC: https://www.thessgac.org/

UCSC Genome Browser data integrator tool: https://genome.ucsc.edu/cgi-bin/hgIntegrator

Supplementary Tables

Supplementary Table 1: Discovery ASD-MVR ($5x10^{-8}<P_{thr}<0.5$)

Variant selection			Intercept		ASD effect		ADHD effect	
G_i	N_{SNPs}	P_{thr}	$\hat{\theta}_{0*}$(SE)	*P*	$\hat{\theta}_{ASD}$(SE)	*P*	$\hat{\theta}_{*ADHD}$(SE)	*P*
ASD (iPSYCH, woADHD)	3‡	$5x10^{-8}$	NA	NA	NA	NA	NA	NA
	5	$5x10^{-7}$	-0.010(0.007)	0.28	0.10(0.065)	0.26	-0.015(0.028)	0.65
	26	$5x10^{-6}$	0.004(0.003)	0.16	-0.003(0.030)	0.93	-0.022(0.024)	0.38
	121	$5x10^{-5}$	0.001(0.001)	0.38	0.010(0.013)	0.44	-0.018(0.014)	0.22
	816	0.0005	$2x10^{-4}(4x10^{-4})$	0.54	0.015(0.005)	0.002	-0.064(0.006)	10^{-8}
	1,973	0.0015	$0.001(2x10^{-4})$	0.008	0.009(0.003)	0.002	-0.029(0.004)	$<10^{-10}$
	5,399	0.005	$5x10^{-4}(10^{-4})$	$3x10^{-4}$	0.010(0.002)	$6x10^{-9}$	-0.028(0.002)	$<10^{-10}$
	35,921	0.05	$3x10^{-4}(4x10^{-5})$	$<10^{-10}$	0.007(0.001)	$<10^{-10}$	-0.022(0.001)	$<10^{-10}$
	62,589	0.1	$2x10^{-4}(3x10^{-5})$	$2x10^{-10}$	0.007(0.001)	$<10^{-10}$	-0.020(0.001)	$<10^{-10}$
	134,210	0.3	$1x\text{-}10^{-4}(2x10^{-5})$	$3x10^{-9}$	$0.007(4x10^{-4})$	$<10^{-10}$	$-0.018(4x10^{-4})$	$<10^{-10}$
	185,632	0.5	$10^{-4}(2x10^{-5})$	$<10^{-10}$	$0.007(4x10^{-4})$	$<10^{-10}$	$-0.017(3x10^{-4})$	$<10^{-10}$

Abbreviations: ADHD, Attention-Deficit/Hyperactivity Disorder; ASD, Autism Spectrum Disorder; iPSYCH, The Lundbeck Foundation Initiative for Integrative Psychiatric Research; N_{SNPs}, number of SNPs; P_{thr}, *P*-value threshold; woADHD, without ADHD

Estimated ASD effect ($\hat{\theta}_{ASD}$) and ADHD effect ($\hat{\theta}_{*ADHD}$) on EA as fitted with ASD-MVR (Supplementary Figure 1a). Sets of independent ASD (G_i) variants were selected from ASD(iPSYCH, woADHD). SNP estimates for ASD ($\hat{\beta}_{ASD}$), ADHD ($\hat{\beta}_{ADHD}$) and EA ($\hat{\beta}_{EA}$) were extracted from ASD(iPSYCH, woADHD), ADHD(iPSYCH) and EA(SSGAC) GWAS statistics respectively. All MVR effects are presented as change in years of schooling per increase in log odds of ASD or ADHD liability. Individual effect estimates, standard errors and corresponding *P*-values (t-statistic, two-sided test) are shown. The multiple-testing-adjusted threshold is $P<0.0023$.

‡ MVR analyses for ASD-related variants ($P_{thr}<5x10^{-8}$) did not converge.

Supplementary Table 2: Discovery ADHD-MVR ($5\text{x}10^{-8}<P_{thr}<0.5$)

Variant selection			Intercept		ADHD effect		ASD effect	
G_j	N_{SNPs}	P_{thr}	$\hat{\theta}_{0\#}$(SE)	P	$\hat{\theta}_{ADHD}$(SE)	P	$\hat{\theta}_{\#ASD}$(SE)	P
ADHD (iPSYCH)	10	$5\text{x}10^{-8}$	-0.006(0.017)	0.76	-0.068(0.16)	0.68	0.12(0.075)	0.16
	22	$5\text{x}10^{-7}$	-0.002(0.006)	0.70	-0.055(0.060)	0.37	0.061(0.044)	0.19
	61	$5\text{x}10^{-6}$	-0.003(0.002)	0.13	-0.048(0.024)	0.054	0.096(0.026)	$5\text{x}10^{-4}$
	242	$5\text{x}10^{-5}$	-0.003(0.001)	0.001	-0.013(0.011)	0.24	0.025(0.012)	0.036
	1,202	0.0005	-0.002($3\text{x}10^{-4}$)	$2\text{x}10^{-6}$	-0.015(0.004)	0.001	0.023(0.005)	$2\text{x}10^{-6}$
	2,717	0.0015	-0.002($2\text{x}10^{-4}$)	$<10^{-10}$	-0.012(0.003)	$4\text{x}10^{-5}$	0.022(0.003)	$<10^{-10}$
	6,781	0.005	-0.001(10^{-4})	$<10^{-10}$	-0.009(0.002)	$5\text{x}10^{-8}$	0.018(0.002)	$<10^{-10}$
	41,334	0.05	-0.001($4\text{x}10^{-5}$)	$<10^{-10}$	-0.009(0.001)	$<10^{-10}$	0.013(0.001)	$<10^{-10}$
	71,015	0.1	-0.001($3\text{x}10^{-5}$)	$<10^{-10}$	-0.009(0.001)	$<10^{-10}$	0.012(0.001)	$<10^{-10}$
	164,083	0.3	$-3\text{x}10^{-4}$($2\text{x}10^{-5}$)	$<10^{-10}$	-0.010($4\text{x}10^{-4}$)	$<10^{-10}$	0.010($3\text{x}10^{-4}$)	$<10^{-10}$
	234,530	0.5	$-2\text{x}10^{-4}$(10^{-5})	$<10^{-10}$	-0.011($4\text{x}10^{-4}$)	$<10^{-10}$	0.009($3\text{x}10^{-4}$)	$<10^{-10}$

Abbreviations: ADHD, Attention-Deficit/Hyperactivity Disorder; ASD, Autism Spectrum Disorder; iPSYCH, The Lundbeck Foundation Initiative for Integrative Psychiatric Research; N_{SNPs}, number of SNPs; P_{thr}, *P*-value threshold; woADHD, without ADHD

Estimated ADHD effect ($\hat{\theta}_{ADHD}$) and ASD effect ($\hat{\theta}_{\#ASD}$) on EA as fitted with ADHD-MVR (Supplementary Figure 1b). Sets of independent ADHD (G_j) genetic variants were selected from ADHD(iPSYCH) GWAS statistics. SNP estimates for ASD ($\hat{\beta}_{ASD}$), ADHD ($\hat{\beta}_{ADHD}$) and EA ($\hat{\beta}_{EA}$) were extracted from ASD(iPSYCH, woADHD), ADHD(iPSYCH) and EA(SSGAC) GWAS statistics respectively. All MVR effects are presented as change in years of schooling per increase in log odds of ASD or ADHD liability. Individual effect estimates, standard errors and corresponding *P*-values (t-statistic, two-sided test) are shown. The multiple-testing-adjusted threshold is $P<0.0023$.

Supplementary Table 3: Discovery ASD-MVR and ADHD-MVR (P_{thr}<0.0015; P_{thr}<0.05), model fit

ASD-MVR											
Variant selection			Intercept		ASD effect		ADHD effect		VIF	MVR to UVR model fit comparison	
G_i	N_{SNPs}	P_{thr}	$\hat{\theta}_{0*}$(SE)	P	$\hat{\theta}_{ASD}$(SE)	P	$\hat{\theta}_{*ADHD}$(SE)	P		ΔR^2 (%)	LRT-χ^2(df=1), P
ASD (iPSYCH, woADHD)	1,973	0.0015	0.001($2x10^{-4}$)	0.008	$-5x10^{-6}$(0.003)	1.00	-		-	-	
			0.001($2x10^{-4}$)	0.008	0.009(0.003)	0.002	-0.029(0.004)	$<10^{-10}$	1.19	3.0	167.06, $<10^{-10}$
	35,921	0.05	$3x10^{-4}$($4x10^{-5}$)	10^{-9}	0.001(0.001)	0.17	-		-	-	
			$3x10^{-4}$($4x10^{-5}$)	$<10^{-10}$	0.007(0.001)	$<10^{-10}$	-0.022(0.001)	$<10^{-10}$	1.11	2.1	1533.8, $<10^{-10}$

ADHD-MVR											
Variant selection			Intercept		ADHD effect		ASD effect		VIF	MVR to UVR model fit comparison	
G_j	N_{SNPs}	P_{thr}	$\hat{\theta}_{0\#}$(SE)	P	$\hat{\theta}_{ADHD}$(SE)	P	$\hat{\theta}_{\#ASD}$(SE)	P		ΔR^2 (%)	LRT-χ^2(df=1), P
ADHD (iPSYCH)	2,717	0.0015	-0.001($2x10^{-4}$)	$<10^{-10}$	-0.004(0.003)	0.16	-		-	-	
			-0.002($2x10^{-4}$)	$<10^{-10}$	-0.012(0.003)	$4x10^{-5}$	0.022(0.003)	$<10^{-10}$	1.18	1.9	154.24, $<10^{-10}$
	41,334	0.05	-0.001($4x10^{-5}$)	$<10^{-10}$	-0.004(0.001)	$<10^{-10}$	-		-	-	
			-0.001($4x10^{-5}$)	$<10^{-10}$	-0.009(0.001)	$<10^{-10}$	0.013(0.001)	$<10^{-10}$	1.11	0.9	744.05, $<10^{-10}$

Abbreviations: ADHD, Attention-Deficit/Hyperactivity Disorder; ASD, Autism Spectrum Disorder; df, degrees of freedom; iPSYCH, The Lundbeck Foundation Initiative for Integrative Psychiatric Research; LRT, likelihood-ratio test; MVR, multivariable regression; N_{SNPs}, number of SNPs; P_{thr}, *P*-value threshold; VIF, variance inflation factor; woADHD, without ADHD; UVR, univariable regression; ΔR^2, difference in Regression R^2 between MVR and UVR

Estimated ASD effect ($\hat{\theta}_{ASD}$) and ADHD effect ($\hat{\theta}_{*ADHD}$) on EA as fitted with ASD-MVR (Figure 2a, Supplementary Figure 1a). Estimated ADHD effect ($\hat{\theta}_{ADHD}$) and ASD effect ($\hat{\theta}_{\#ASD}$) on EA as fitted with ADHD-MVR (Figure 2b, Supplementary Figure 1b). Sets of independent ASD (G_i) and ADHD (G_j) genetic variants were selected from ASD(iPSYCH, woADHD) and ADHD(iPSYCH) GWAS statistics respectively and are shown for two *P*-value thresholds (P_{thr}<0.0015, P_{thr}<0.05). SNP estimates for ASD ($\hat{\beta}_{ASD}$), ADHD ($\hat{\beta}_{ADHD}$) and EA ($\hat{\beta}_{EA}$) were extracted from ASD(iPSYCH, woADHD), ADHD(iPSYCH) and EA(SSGAC) GWAS statistics respectively. All MVR effects are presented as change in years of schooling per increase in log odds of ASD or ADHD liability. Individual effect estimates, standard errors and corresponding *P*-values (t-statistic, two-sided test) are shown. The multiple-testing-adjusted threshold is *P*<0.0023. . The model fit of MVRs and UVRs was compared with likelihood-ratio tests (LRTs).

Supplementary Table 4: Discovery ASD-MVR and ADHD-MVR (P_{thr}<0.0015; P_{thr}<0.05), concordant variants

ASD-MVR

Variant selection			Intercept		ASD effect		ADHD effect		VIF	MVR to UVR model fit comparison: ASD UVR		ADHD UVR	
Concordant variants G_i	N_{SNPs}	P_{thr}	$\hat{\theta}_{0*}$(SE)	*P*	$\hat{\theta}_{ASD}$(SE)	*P*	$\hat{\theta}_{*ADHD}$(SE)	*P*		ΔR^2(%)	LRT-χ^2(df=1), *P*	ΔR^2(%)	LRT-χ^2(df=1), *P*
ASD (iPSYCH, woADHD)	1,716	0.0015	$4x10^{-4}(2x10^{-4})$	0.15	0.001(0.003)	0.76	-		-	-			
			$0.001(2x10^{-4})$	$4x10^{-10}$	-		-0.018(0.004)	$9x10^{-6}$	-	-			
			$4x10^{-4}(2x10^{-4})$	0.12	0.011(0.003)	0.001	-0.026(0.005)	$5x10^{-8}$	1.40	1.7	79.67, $4x10^{-8}$	0.6	27.06, 0.001
	28,086	0.05	$3x10^{-5}(5x10^{-5})$	0.49	0.001(0.001)	0.11	-		-	-			
			$5x10^{-4}(3x10^{-5})$	$<1x10^{-10}$			-0.014(0.001)	$<10^{-10}$	-	-			
			$10^{-4}(5x10^{-5})$	0.054	0.009(0.001)	$<10^{-10}$	-0.020(0.001)	$<10^{-10}$	1.36	1.1	579.37, $<10^{-10}$	0.4	203.75, $<10^{-10}$

ADHD-MVR

Variant selection			Intercept		ADHD effects		ASD effect		VIF	MVR to UVR model fit comparison: ADHD UVR		ASD UVR	
Concordant variants G_j	N_{SNPs}	P_{thr}	$\hat{\theta}_{0\#}$(SE)	*P*	$\hat{\theta}_{ADHD}$(SE)	*P*	$\hat{\theta}_{\#ASD}$(SE)	*P*		ΔR^2(%)	LRT-χ^2(df=1), *P*	ΔR^2(%)	LRT-χ^2(df=1), *P*
ADHD (iPSYCH)	2,382	0.0015	$-0.001(2x10^{-4})$	$8x10^{-10}$	-0.004(0.003)	0.16	-		-	-			
			$-0.002(1x10^{-4})$	$<1x10^{-10}$	-		0.014(0.003)	$3x10^{-5}$	-	-			
			$-0.001(2x10^{-4})$	$2x10^{-10}$	-0.013(0.003)	$4x10^{-5}$	0.022(0.004)	10^{-8}	1.36	1.3	92.59, 10^{-8}	0.7	48.75, $4x10^{-5}$
	32,176	0.05	$-4x10^{-4}(5x10^{-5})$	$<10^{-10}$	-0.005(0.001)	$3x10^{-10}$	-		-	-			
			$-9x10^{-4}(3x10^{-5})$	$<1x10^{-10}$	-		$0.007(9x10^{-4})$	$<10^{-10}$	-	-			
			$-0.001(5x10^{-5})$	$<10^{-10}$	-0.011(0.001)	$<10^{-10}$	0.013(0.001)	$<10^{-10}$	1.36	0.5	339.16, $<10^{-10}$	0.5	290.13, $<10^{-10}$

Abbreviations: ADHD, Attention-Deficit/Hyperactivity Disorder; ASD, Autism Spectrum Disorder; df, degrees of freedom; iPSYCH, The Lundbeck Foundation Initiative for Integrative Psychiatric Research; LRT, likelihood-ratio test; MVR, multivariable regression; N_{SNPs}, number of SNPs; P_{thr}, *P*-value threshold; VIF, variance inflation factor; woADHD, without ADHD; UVR, univariable regression; ΔR^2, difference in Regression R^2 between UVR and MVR

Estimated ASD effect ($\hat{\theta}_{ASD}$) and ADHD effect ($\hat{\theta}_{*ADHD}$) on EA as fitted with ASD-MVR (Supplementary Figure 1a). Estimated ADHD effect ($\hat{\theta}_{ADHD}$) and ASD effect ($\hat{\theta}_{\#ASD}$) on EA as fitted with ADHD-MVR (Supplementary Figure 1b). Sets of independent ASD (G_i) and ADHD (G_j) genetic variants were selected at two *P*-value thresholds (P_{thr}<0.0015, P_{thr}<0.05) from ASD(iPSYCH, woADHD) and ADHD(iPSYCH) GWAS statistics respectively and restricted to SNPs with concordant allelic effects. SNP estimates for ASD ($\hat{\beta}_{ASD}$), ADHD ($\hat{\beta}_{ADHD}$) and EA ($\hat{\beta}_{EA}$) were extracted from ASD(iPSYCH, woADHD), ADHD(iPSYCH) and EA(SSGAC) GWAS statistics respectively. All MVR effects are presented as change in years of schooling per increase in log odds of ASD or ADHD liability. Individual effect estimates, standard errors and corresponding *P*-values (t-statistic, two-sided test) are shown. The multiple-testing-adjusted threshold is *P*<0.0023. The model fit of MVRs and UVRs was compared with likelihood-ratio tests (LRTs).

Supplementary Table 5: Genetic correlations among psychiatric disorders

Sample 1	Sample 2	r_g (SE)	*P*
ASD(iPSYCH, woADHD)	ASD(PGC)	0.84(0.11)	$<10^{-10}$
	ADHD(iPSYCH)	0.30 (0.06)	$2x10^{-7}$
	MDD(PGC)	0.43 (0.05)	$<10^{-10}$
	SCZ(PGC)	0.21 (0.06)	10^{-4}
	BD(PGC)	0.18 (0.06)	0.001
ASD(PGC)	ADHD(iPSYCH)	0.04(0.08)	0.61
	MDD(PGC)	0.12(0.05)	0.017
	SCZ(PGC)	0.20(0.06)	0.001
	BD(PGC)	0.12(0.06)	0.063
ADHD(iPSYCH)	MDD(PGC)	0.55 (0.04)	$<10^{-10}$
	SCZ(PGC)	0.12 (0.04)	0.004
	BD(PGC)	0.12 (0.05)	0.007

Abbreviations: ADHD, Attention-Deficit/Hyperactivity Disorder; ASD, Autism Spectrum Disorder; BD, Bipolar Disorder; iPSYCH, The Lundbeck Foundation Initiative for Integrative Psychiatric Research; MDD; Major Depressive Disorder; PGC, Psychiatric Genomics Consortium; r_g, genetic correlation; SCZ, Schizophrenia; woADHD, without ADHD

Genetic correlations (r_g) among psychiatric disorder samples were estimated using summary statistics and unconstrained LD score correlation[1]. Individual genetic correlation estimates, standard errors and corresponding *P*-values (Z-statistic, two-sided test) are shown. The multiple testing threshold was *P*<0.002.

Supplementary Table 6: Genetic correlations of psychiatric disorders with educational attainment

Sample 1	Sample 2	r_g (SE)	*P*
EA(SSGAC)	ASD(iPSYCH, woADHD)	0.23(0.03)	$<10^{-10}$
	ASD(PGC)	0.28(0.03)	$<10^{-10}$
	ADHD(iPSYCH)	-0.49(0.03)	$<10^{-10}$
	MDD(PGC)	-0.22 (0.03)	$<10^{-10}$
	SCZ(PGC)	0.07 (0.02)	0.003
	BD(PGC)	0.18 (0.02)	$<10^{-10}$
	ASD(iPSYCH, woADHD) + ADHD(iPSYCH)	-0.23(0.03)	$<10^{-10}$
	ASD(PGC) + ADHD(iPSYCH)	-0.38(0.03)	$<10^{-10}$
	ASD(iPSYCH, woADHD) + MDD(PGC)	-0.13(0.03)	$4x10^{-7}$
	ADHD(iPSYCH) + BD(PGC)	-0.18(0.03)	$<10^{-10}$

Abbreviations: ADHD, Attention-Deficit/Hyperactivity Disorder; ASD, Autism Spectrum Disorder; BD, Bipolar Disorder; EA; educational attainment; iPSYCH, The Lundbeck Foundation Initiative for Integrative Psychiatric Research; MDD, Major Depressive Disorder; PGC, Psychiatric Genomics Consortium; r_g, genetic correlation; SCZ, Schizophrenia; SSGAC, Social Science Genetic Association Consortium; woADHD, without ADHD

Genetic correlations (r_g) were estimated using summary statistics and unconstrained LD score correlation[1]. Individual genetic correlation estimates, standard errors and corresponding *P*-values (Z-statistic, two-sided test) are shown. The multiple testing threshold was *P*<0.002.

Supplementary Table 7: Follow-up ASD-MVR and ADHD-MVR (P_{thr}<0.0015; P_{thr}<0.05), analyses with ASD(PGC) SNP estimates (independent variable)

ASD-MVR

Variant selection			Intercept		ASD effects		ADHD effect		VIF	MVR to UVR model fit comparison	
G_i	N_{SNPs}	P_{thr}	$\hat{\theta}_{0*}$(SE)	P	$\hat{\theta}_{ASD}$(SE)	P	$\hat{\theta}_{*ADHD}$(SE)	P		ΔR^2(%)	LRT-χ^2(df=1), P
ASD (iPSYCH, ASDwoADHD) variants with ASD(PGC) estimates	1,886	0.0015	$-6\times10^{-5}(10^{-4})$	0.69	0.010(0.003)	0.004	-		-	-	
			$-5\times10^{-5}(10^{-4})$	0.70	0.010(0.003)	0.003	-0.003(0.003)	0.31	1.00	0.05	2.98, 0.31
	33,845	0.05	$-10^{-5}(3\times10^{-5})$	0.61	0.005(0.001)	$<10^{-10}$	-		-	-	
			$-9\times10^{-6}(3\times10^{-5})$	0.76	0.005(0.001)	$<10^{-10}$	-0.007(0.001)	$<10^{-10}$	1.00	0.41	285.63, $<10^{-10}$

ADHD-MVR

Variant selection			Intercept		ADHD effect		ASD effect		VIF	MVR to UVR model fit comparison	
G_j	N_{SNPs}	P_{thr}	$\hat{\theta}_{0\#}$(SE)	P	$\hat{\theta}_{ADHD}$(SE)	P	$\hat{\theta}_{\#ASD}$(SE)	P		ΔR^2(%)	LRT-χ^2(df=1), P
ADHD (iPSYCH)	2,627	0.0015	$-0.001(2\times10^{-4})$	$<10^{-10}$	-0.004(0.003)	0.14	-		-	-	
			$-0.001(2\times10^{-4})$	$<10^{-10}$	-0.004(0.003)	0.13	0.002(0.002)	0.35	1.00	0.03	2.62, 0.35
	38,656	0.05	$-0.001(4\times10^{-5})$	$<10^{-10}$	-0.005(0.001)	$<10^{-10}$	-		-	-	
			$-0.001(4\times10^{-5})$	$<10^{-10}$	-0.005(0.001)	$<10^{-10}$	$0.003(4\times10^{-4})$	$<10^{-10}$	1.00	0.16	123.59, $<10^{-10}$

Abbreviations: ADHD, Attention-Deficit/Hyperactivity Disorder; ASD, Autism Spectrum Disorder; df, degrees of freedom; iPSYCH, The Lundbeck Foundation Initiative for Integrative Psychiatric Research; LRT, likelihood-ratio test; MVR, multivariable regression; N_{SNPs}, number of SNPs; PGC, Psychiatric Genomics Consortium; P_{thr}, P-value threshold; VIF, variance inflation factor; UVR, univariable regression; ΔR^2, difference in Regression R^2 between MVR and UVR

Estimated ASD effect ($\hat{\theta}_{ASD}$) and ADHD effect ($\hat{\theta}_{*ADHD}$) on EA as fitted with ASD-MVR (Supplementary Figure 1c). Estimated ADHD effect ($\hat{\theta}_{ADHD}$) and ASD effect ($\hat{\theta}_{\#ASD}$) on EA as fitted with ADHD-MVR (Supplementary Figure 1d). Sets of independent ASD (G_i) and ADHD (G_j) genetic variants were selected from ASD(iPSYCH, woADHD) and ADHD(iPSYCH) GWAS statistics respectively and are shown for two P-value thresholds (P_{thr}<0.0015, P_{thr}<0.05). SNP estimates for ASD ($\hat{\beta}_{ASD}$), ADHD ($\hat{\beta}_{ADHD}$) and EA ($\hat{\beta}_{EA}$) were extracted from ASD(PGC), ADHD(iPSYCH) and EA(SSGAC) GWAS statistics respectively. All MVR effects are presented as change in years of schooling per increase in log odds of ASD or ADHD liability. Individual effect estimates, standard errors and corresponding P-values (t-statistic, two-sided test) are shown. The multiple-testing-adjusted threshold is P<0.0125. The model fit of MVRs and UVRs was compared with likelihood-ratio tests (LRTs).

Supplementary Table 8: Follow-up ASD-MVR and ADHD-MVR (P_{thr}<0.0015; P_{thr}<0.05) with ASD(PGC) SNP estimates (independent variable), concordant variants

ASD-MVR

Variant selection			Intercept		ASD effect		ADHD effect		VIF	MVR to UVR model fit comparison: ASD UVR		ADHD UVR	
Concordant variants G_i	N_{SNPs}	P_{thr}	$\hat{\theta}_{0*}$(SE)	*P*	$\hat{\theta}_{ASD}$(SE)	*P*	$\hat{\theta}_{*ADHD}$(SE)	*P*		ΔR^2(%)	LRT-χ^2 (df=1), *P*	ΔR^2(%)	LRT-χ^2 (df=1), *P*
ASD (iPSYCH, woADHD) variants with ASD(PGC) estimates	923	0.0015	2x10^{-4}(2x10^{-4})	0.29	0.009(0.005)	0.061	-		-	-			
			0.001(2x10^{-4})	6x10^{-9}	-		-0.026(0.006)	9x10^{-6}	-	-			
			0.001(3x10^{-4})	2x10^{-4}	0.015(0.005)	0.003	-0.030(0.006)	6x10^{-7}	1.05	2.7	78.55, 5x10^{-7}	0.9	27.5, 0.003
	17,506	0.05	-2x10^{-4}(4x10^{-5})	4x10^{-6}	0.005(9x10^{-4})	7x10^{-9}	-		-	-			
			2x10^{-4}(4x10^{-5})	6x10^{-9}	-		-0.011(0.001)	<1x10^{-10}	-	-			
			6x10^{-5}(5x10^{-5})	0.19	0.007(0.001)	<1x10^{-10}	-0.013(0.001)	<1x10^{-10}	1.05	0.5	194.9, <10^{-10}	0.3	126.2, <10^{-10}

ADHD-MVR

Variant selection			Intercept		ADHD effects		ASD effect		VIF	MVR to UVR model fit comparison: ADHD UVR		ASD UVR	
Concordant variants G_j	N_{SNPs}	P_{thr}	$\hat{\theta}_{0\#}$(SE)	*P*	$\hat{\theta}_{ADHD}$(SE)	*P*	$\hat{\theta}_{\#ASD}$(SE)	*P*		ΔR^2(%)	LRT-χ^2 (df=1), *P*	ΔR^2(%)	LRT-χ^2 (df=1), *P*
ADHD (iPSYCH)	1,362	0.0015	-0.001(3x10^{-4})	3x10^{-4}	-0.008(0.004)	0.022	-		-	-			
			-0.001(2x10^{-4})	<1x10^{-10}	-		-0.005(0.004)	0.19	-	-			
			-0.001(3x10^{-4})	4x10^{-4}	-0.008(0.004)	0.053	-0.002(0.004)	0.67	1.19	0.01	0.52, 0.67	0.3	10.8, 0.05
	19,615	0.05	-4x10^{-4}(6x10^{-5})	1x10^{-10}	-0.006(0.001)	9x10^{-10}	-		-	-			
			-0.001(4x10^{-5})	<1x10^{-10}	-		2x10^{-4}(0.001)	0.80	-	-			
			-4x10^{-4}(6x10^{-5})	<1x10^{-10}	-0.008(0.001)	<1x10^{-10}	0.003(0.001)	0.003	1.19	0.05	18.15, 0.003	0.2	94.9, <10^{-10}

Abbreviations: ADHD, Attention-Deficit/Hyperactivity Disorder; ASD, Autism Spectrum Disorder; df, degrees of freedom; iPSYCH, The Lundbeck Foundation Initiative for Integrative Psychiatric Research; LRT, likelihood-ratio test; MVR, multivariable regression; N_{SNPs}, number of SNPs; P_{thr}, *P*-value threshold; VIF, variance inflation factor; woADHD, without ADHD; UVR, univariable regression; ΔR^2, difference in Regression R^2 between UVR and MVR.

Estimated ASD effect ($\hat{\theta}_{ASD}$) and ADHD effect ($\hat{\theta}_{*ADHD}$) on EA as fitted with ASD-MVR (Supplementary Figure 1c). Estimated ADHD effect ($\hat{\theta}_{ADHD}$) and ASD effect ($\hat{\theta}_{\#ASD}$) on EA as fitted with ADHD-MVR (Supplementary Figure 1d). Sets of independent ASD (G_i) and ADHD (G_j) genetic variants were selected at two *P*-value thresholds (P_{thr}<0.0015, P_{thr}<0.05) from ASD(iPSYCH, woADHD) and ADHD(iPSYCH) GWAS statistics respectively. SNP estimates for ASD ($\hat{\beta}_{ASD}$), ADHD ($\hat{\beta}_{ADHD}$) and EA ($\hat{\beta}_{EA}$) were extracted from ASD(PGC), ADHD(iPSYCH) and EA(SSGAC) GWAS statistics respectively and analyses were restricted to concordant ASD and ADHD SNP estimates. All MVR effects are presented as change in years of schooling per increase in log odds of ASD or ADHD liability. Individual effect estimates, standard errors and corresponding *P*-values (t-statistic, two-sided test) are shown. The multiple-testing-adjusted threshold is *P*<0.0125. The model fit of MVRs and UVRs was compared with likelihood-ratio tests (LRTs).

Supplementary Table 9: ASD-MVR and ADHD-MVR with variants from LD blocks with high posterior probability for pleiotropy

ASD-MVR

Variant selection		Intercept		ASD effects		ADHD effect		VIF	MVR to UVR model fit comparison	
G_i	N_{SNPs}	$\hat{\theta}_{0*}$(SE)	*P*	$\hat{\theta}_{ASD}$(SE)	*P*	$\hat{\theta}_{*ADHD}$(SE)	*P*		ΔR^2(%)	LRT-χ^2(df=1), *P*
ASD (iPSYCH, woADHD)	465	0.001($5x10^{-4}$)	0.005	0.004(0.009)	0.67	-		-	-	
		0.001($5x10^{-4}$)	0.004	0.027(0.010)	0.009	-0.042(0.008)	$2x10^{-7}$	1.22	5.6	95.67, $2x10^{-7}$

ADHD-MVR

Variant selection		Intercept		ADHD effect		ASD effect		VIF	MVR to UVR model fit comparison	
G_j	N_{SNPs}	$\hat{\theta}_{0\#}$(SE)	*P*	$\hat{\theta}_{ADHD}$(SE)	*P*	$\hat{\theta}_{\#ASD}$(SE)	*P*		ΔR^2(%)	LRT-χ^2(df=1), *P*
ADHD (iPSYCH)	481	$6x10^{-5}$($5x10^{-5}$)	0.90	-0.015(0.009)	0.099	-		-	-	
		$7x10^{-6}$($5x10^{-4}$)	0.99	-0.037(0.010)	$2x10^{-4}$	0.043(0.008)	$2x10^{-8}$	1.18	6.3	112.17, $1x10^{-8}$

Abbreviations: ADHD, Attention-Deficit/Hyperactivity Disorder; ASD, Autism Spectrum Disorder; df, degrees of freedom; LRT, likelihood-ratio test; MVR, multivariable regression; N_{SNPs}, number of SNPs; PPA; posterior probability; VIF, variance inflation factor; UVR, univariable regression; ΔR^2, difference in Regression R^2 between UVR and MVR

Estimated ASD effect ($\hat{\theta}_{ASD}$) and ADHD effect ($\hat{\theta}_{*ADHD}$) on EA as fitted with ASD-MVR (Supplementary Figure 1e). Estimated ADHD effect ($\hat{\theta}_{ADHD}$) and ASD effect ($\hat{\theta}_{\#ASD}$) on EA as fitted with ADHD-MVR (Supplementary Figure 1f). Approximately independent LD blocks with evidence for pleiotropy or high-LD co-localisation were identified using gwas-pw^2 (posterior probability > 0.9) and ASD(iPSYCH, woADHD) and ADHD(iPSYCH) GWAS statistics. Within each LD block, independent ASD- and ADHD-related variants (LD-r^2<0.25 within ±500 kb) were identified using information from ASD(iPSYCH, woADHD) and ADHD(iPSYCH). SNP estimates for ASD ($\hat{\beta}_{ASD}$), ADHD ($\hat{\beta}_{ADHD}$) and EA ($\hat{\beta}_{EA}$) were extracted from ASD(iPSYCH, woADHD), ADHD(iPSYCH) and EA(SSGAC) GWAS statistics respectively. All MVR effects are presented as change in years of schooling per increase in log odds of ASD or ADHD liability. Individual effect estimates, standard errors and corresponding *P*-values (t-statistic, two-sided test) are shown. The multiple-testing-adjusted threshold is *P*<0.0023.The model fit of MVRs and UVRs was compared with likelihood-ratio tests (LRTs).

Supplementary Table 10: Identification of single variants using conditional *P*-value thresholding: ASD-MVR

Variant selection					Intercept		ASD effect		ADHD effect	
$G_{i\|j}$	N_{SNPs}	% of G_i	P_{thr} ASD	P_{thr} ADHD	$\hat{\theta}_{0*}$(SE)	*P*	$\hat{\theta}_{ASD}$(SE)	*P*	$\hat{\theta}_{*ADHD}$(SE)	*P*
ASD (iPSYCH, woADHD)	83	4.21	0.0015	0.0015	-0.003(0.001)	0.010	0.15(0.025)	10^{-7}	-0.15(0.025)	10^{-7}
	134	6.79		0.005	-0.002(0.001)	0.15	0.12(0.022)	$6x10^{-7}$	-0.13(0.024)	$2x10^{-7}$
	393	19.92		0.05	-0.001(0.001)	0.28	0.045(0.009)	$4x10^{-7}$	-0.063(0.011)	$7x10^{-9}$
	536	27.17		0.1	$-4x10^{-4}(5x10^{-4})$	0.40	0.040(0.007)	$2x10^{-8}$	-0.059(0.009)	$<10^{-10}$
	805	40.80		0.3	$5x10^{-4}(4x10^{-4})$	0.21	0.021(0.005)	$4x10^{-5}$	-0.044(0.006)	$<10^{-10}$
	914	46.33		0.5	$0.001(3x10^{-4})$	0.12	0.017(0.004)	10^{-4}	-0.039(0.006)	$<10^{-10}$
	1973^{+}	100		1	$0.001(2x10^{-4})$	0.008	0.009(0.003)	0.002	-0.029(0.004)	$<10^{-10}$

Abbreviations: ADHD, Attention-Deficit/Hyperactivity Disorder; ASD, Autism Spectrum Disorder; iPSYCH, The Lundbeck Foundation Initiative for Integrative Psychiatric Research; N_{SNPs}, number of SNPs; P_{thr}, *P*-value threshold; woADHD, without ADHD; $^{+}$Discovery ASD variant set (P_{thr} ASD<0.0015)

Estimated ASD effect ($\hat{\theta}_{ASD}$) and ADHD effect ($\hat{\theta}_{*ADHD}$) on EA as fitted with ASD-MVR (Figure 4a, Supplementary Figure 1g) using six SNP subsets ($G_{i|j}$) fulfilling joint ASD and ADHD selection criteria. Independent genetic variants were selected from ASD(iPSYCH, woADHD) and ADHD(iPSYCH) GWAS statistics. Among ASD variants of the discovery variant set ($^{+}$, P_{thr}<0.0015), we identified those SNPs, which were also associated with ADHD (six thresholds: 0.0015≤ADHD-P_{thr}<0.5). Variants were identified as tagged, if SNPs were within 500kb and LD-r^2≥0.6. SNP estimates for ASD ($\hat{\beta}_{ASD}$), ADHD ($\hat{\beta}_{ADHD}$) and EA ($\hat{\beta}_{EA}$) were extracted from ASD(iPSYCH, woADHD), ADHD(iPSYCH) and EA(SSGAC) GWAS statistics respectively. All MVR effects are presented as change in years of schooling per increase in log odds of ASD or ADHD liability. Individual effect estimates, standard errors and corresponding *P*-values (t-statistic, two-sided test) are shown. The multiple-testing-adjusted threshold is *P*<0.0023.

Supplementary Table 11: Identification of single variants using conditional *P*-value thresholding: ADHD-MVR

Variant sets ($G_{j\|i}$)					Intercept		ADHD effect		ASD effect	
$G_{j\|i}$	N_{SNPs}	% of G_j	P_{thr} ADHD	P_{thr} ASD	$\hat{\theta}_{0\#}$(SE)	*P*	$\hat{\theta}_{ADHD}$(SE)	*P*	$\hat{\theta}_{\#ASD}$(SE)	*P*
ADHD (iPSYCH)	83	3.05	0.0015	0.0015	-0.003(0.001)	0.038	-0.10(0.026)	$2x10^{-4}$	0.12(0.022)	$9x10^{-7}$
	145	5.34		0.005	-0.002(0.001)	0.050	-0.070(0.022)	0.002	0.086(0.019)	10^{-5}
	473	17.41		0.05	-0.001(0.001)	0.044	-0.026(0.010)	0.010	0.032(0.010)	0.001
	638	23.48		0.1	-0.001($4x10^{-4}$)	0.047	-0.023(0.008)	0.003	0.026(0.008)	0.001
	937	34.49		0.3	-0.001($3x10^{-4}$)	0.003	-0.018(0.005)	$5x10^{-4}$	0.023(0.006)	$5x10^{-5}$
	1020	37.54		0.5	-0.001($3x10^{-4}$)	0.005	-0.017(0.005)	$3x10^{-4}$	0.018(0.005)	$3x10^{-4}$
	2717$^+$	100		1	-0.002($2x10^{-4}$)	$<10^{-10}$	-0.012(0.003)	$4x10^{-5}$	0.022(0.003)	$<10^{-10}$

Abbreviations: ADHD, Attention-Deficit/Hyperactivity Disorder; ASD, Autism Spectrum Disorder; iPSYCH, The Lundbeck Foundation Initiative for Integrative Psychiatric Research; N_{SNPs}, number of SNPs; P_{thr}, *P*-value threshold; woADHD, without ADHD; $^+$Discovery ADHD variant set (P_{thr} ADHD<0.0015)

Estimated ADHD effect ($\hat{\theta}_{ADHD}$) and ASD effect ($\hat{\theta}_{\#ASD}$) on EA as fitted with ADHD-MVR (Figure 4b, Supplementary Figure 1h) using SNP subsets ($G_{j|i}$) fulfilling joint ASD and ADHD selection criteria. Independent genetic variants were selected from ASD(iPSYCH, woADHD) and ADHD(iPSYCH) GWAS statistics. Among ADHD variants of the discovery variant set ($^+$, P_{thr}<0.0015), we identified those SNPs, which were also associated with ASD (six thresholds: 0.0015≤ ASD-P_{thr} <0.5). Variants were identified as tagged, if SNPs were within 500kb and LD-r^2≥0.6. SNP estimates for ASD ($\hat{\beta}_{ASD}$), ADHD ($\hat{\beta}_{ADHD}$) and EA ($\hat{\beta}_{EA}$) were extracted from ASD(iPSYCH, woADHD), ADHD(iPSYCH) and EA(SSGAC) GWAS statistics respectively. All MVR effects are presented as change in years of schooling per increase in log odds of ASD or ADHD liability. Individual effect estimates, standard errors and corresponding *P*-values (t-statistic, two-sided test) are shown. The multiple-testing-adjusted threshold is *P*<0.0023..

Supplementary Table 12: Permutation analysis

ASD-MVR				
Variant selection			Empirical *P*-value (SE)	
P_{thr} ASD	P_{thr} ADHD	N_{SNPs}	ASD effect ($\hat{\boldsymbol{\theta}}_{ASD}$)	ADHD effect ($\hat{\boldsymbol{\theta}}_{*ADHD}$)
0.0015	0.0015	83	$<10^{-4}(<10^{-4})$	$7x10^{-4}(3x10^{-4})$

ADHD-MVR				
Variant selection			Empirical *P*-value (SE)	
P_{thr} ASD	P_{thr} ADHD	N_{SNPs}	ADHD effect ($\hat{\boldsymbol{\theta}}_{ADHD}$)	ASD effect ($\hat{\boldsymbol{\theta}}_{\#ASD}$)
0.0015	0.0015	83	$9x10^{-4}(3x10^{-4})$	$2x10^{-4}(10^{-4})$

Abbreviations: ADHD, Attention-Deficit/Hyperactivity Disorder; ASD, Autism Spectrum Disorder; iPSYCH, The Lundbeck Foundation Initiative for Integrative Psychiatric Research; N_{SNPs}, number of SNPs; P_{thr}, *P*-value threshold; woADHD, without ADHD

For each permutation, 83 independent SNPs were either randomly selected from G_i (P_{thr}<0.0015), for ASD-MVR, or G_j (P_{thr}<0.0015), for ADHD-MVR, based on ASD(iPSYCH, woADHD) and ADHD(iPSYCH) GWAS statistics, respectively. Corresponding SNP estimates for ASD ($\hat{\beta}_{ASD}$), ADHD ($\hat{\beta}_{ADHD}$) and EA ($\hat{\beta}_{EA}$) were extracted from ASD(iPSYCH, woADHD), ADHD(iPSYCH) and EA(SSGAC) GWAS statistics, respectively. For both, empirical ASD-MVR and ADHD-MVR analyses, 10,000 permutations were carried out, in total.

Supplementary Table 13: Gene-set enrichment analysis

Category	GeneSet	N_{genes}	$N_{overlap}$	$P_{adjusted}$	Genes
GO_bp	GO_ANATOMICAL_STRUCTURE_FORMATION_ INVOLVED_IN_MORPHOGENESIS	1143	9	0.047	*PIK3R3, VAV3, KIF26B, TMOD3, EDAR, TGFBR2, RHOA, EXOC4, TEK*
GO_bp	GO_RESPONSE_TO_HORMONE	968	8	0.047	*PIK3R3, CHRM3, MYO5A, ATP6V1C2, TGFBR2, RHOA, OSTN, TEK*
GO_bp	GO_RESPONSE_TO_ENDOGENOUS_ STIMULUS	1627	10	0.047	*PIK3R3, CHRM3, MYO5A, ATP6V1C2, ERBB4, VWC2L, TGFBR2, RHOA, OSTN, TEK*
GO_bp	GO_ENZYME_LINKED_RECEPTOR_ PROTEIN_SIGNALING_PATHWAY	1030	8	0.047	*PIK3R3, VAV3, ATP6V1C2, ERBB4, VWC2L, TGFBR2, RHOA, TEK*
GO_bp	GO_CELLULAR_RESPONSE_TO_ ENDOGENOUS_STIMULUS	1366	9	0.047	*PIK3R3, CHRM3, MYO5A, ATP6V1C2, ERBB4, VWC2L, TGFBR2, RHOA, OSTN*
GO_bp	GO_POSITIVE_REGULATION_OF_ PHOSPHORUS_METABOLIC_PROCESS	1069	8	0.047	*PIK3R3, VAV3, RANBP2, EDAR, ERBB4, TGFBR2, RHOA, TEK*
GO_bp	GO_ORGANOPHOSPHATE_METABOLIC_ PROCESS	529	6	0.047	*PIK3R3, VAV3, FMO2, IP6K2, RHOA, TEK*
GWAS Catalog	Intelligence (MTAG)	313	6	$4.8x10^{-3}$	*SCG3, ERBB4, BSN, CACNA2D2, LINC00461, EXOC4*
GWAS Catalog	Morning person	201	5	$5.0x10^{-3}$	*IP6K2, RHOA, BSN, CACNA2D2, CSMD1*
GWAS Catalog	General cognitive ability	242	5	$8.1x10^{-3}$	*IP6K2, RHOA, BSN, LINC00461, EXOC4*
GO_cc	GO_GLUTAMATERGIC_SYNAPSE	348	5	0.024	*CHRM3, MYO5A, ERBB4, RHOA, BSN*
GO_cc	GO_SYNAPSE	1164	8	0.024	*CHRM3, CACNA1C, MYO5A, ERBB4, VWC2L, RHOA, BSN, EXOC4*
GO_cc	GO_POSTSYNAPSE	608	6	0.024	*CHRM3, CACNA1C, MYO5A, ERBB4, RHOA, BSN*
GO_cc	GO_RECEPTOR_COMPLEX	397	5	0.024	*PKD2L1, ERBB4, VWC2L, TGFBR2, TEK*
GO_cc	GO_CELL_JUNCTION	1263	8	0.024	*CHRM3, CACNA1C, TMOD3, VWC2L, IP6K2, RHOA, BSN, TEK*
microRNA_targets	TTTGCAC_MIR19A_MIR19B	515	7	$7.7x10^{-4}$	*PIK3R3, KIAA1217, CACNA1C, ERBB4, TGFBR2, BSN, CSMD1*
microRNA_targets	ACCAAAG_MIR9	500	5	0.028	*PIK3R3, VAV3, KIAA1217, RANBP2, AUH*

Gene-set enrichment (>5 overlapping genes, FDR-adjusted *P*-values) was conducted with respect to pre-defined gene-sets derived from the Molecular Signature Database (v7.0), WikiPathways (v.20191010), or the GWAS Catalog (v.e96_r2019-09-24). Enrichment analysis was carried out with MAGMA[3] (v1.08) using a one-sided hypergeometric test, as implemented in FUMA[4] software (v1.3.6a), by mapping identified genes for the 83 loci to unique Ensembl IDs (v92). The False Discovery Rate (FDR) was controlled using the Benjamini Hochberg procedure (FUMA, v1.3.6a).

Supplementary Table 14: Sample description for MDD, SCZ, BD and general intelligence

Source	GWAS	Consortium	Imputation reference panel	N
Clinical sample	MDD[5]	PGC	1000 Genomes multi-ancestry	173,005 (cases=59,851)
	SCZ[6]	PGC	1000 Genomes phase 3	65,967 (cases=33,426)
	BD[6]	PGC	1000 Genomes phase 3	41,653 (cases=20,129)
Population sample	Intelligence[7]	CTG lab	HRC[#]	279,930

[#] Predominantly HRC, see Savage et al.[7]

Abbreviations: BD, Bipolar Disorder; CTG, Complex Trait Genetics lab; HRC, Haplotype Reference Consortium; MDD, Major Depressive Disorder; PGC, Psychiatric Genomics Consortium; SCZ, Schizophrenia

All individuals were of European descent.

Supplementary Table 15: Specificity analyses related to general intelligence (dependent variable) using ASD-MVR and ADHD-MVR (P_{thr}<0.0015; P_{thr}<0.05)

ASD-MVR

Variant selection			Intercept		ASD effect		ADHD effect		VIF	MVR to UVR model fit comparison	
G_i	N_{SNPs}	P_{thr}	$\hat{\theta}_{0*}$(SE)	*P*	$\hat{\theta}_{ASD}$(SE)	*P*	$\hat{\theta}_{*ADHD}$(SE)	*P*		ΔR^2 (%)	LRT-χ^2(df=1), *P*
ASD (iPSYCH, woADHD)	1,904	0.0015	0.001(3x10^{-4})	0.01	-0.002(0.004)	0.59	-		-		-
			0.001(3x10^{-4})	0.008	0.008(0.004)	0.05	-0.033(0.005)	5x10^{-10}	1.18	2.02	79.80, 4x10^{-10}
	34,626	0.05	4x10^{-4}(7x10^{-5})	5x10^{-9}	5x10^{-5}(0.001)	0.96	-		-		-
			4x10^{-4}(7x10^{-5})	5x10^{-10}	0.006(0.001)	2x10^{-8}	-0.021(0.001)	<10^{-10}	1.11	0.91	509.13, <10^{-10}

ADHD-MVR

Variant selection			Intercept		ADHD effect		ASD effect		VIF	MVR to UVR model fit comparison	
G_j	N_{SNPs}	P_{thr}	$\hat{\theta}_{0\#}$(SE)	*P*	$\hat{\theta}_{ADHD}$(SE)	*P*	$\hat{\theta}_{\#ASD}$(SE)	*P*		ΔR^2 (%)	LRT-χ^2(df=1), *P*
ADHD (iPSYCH)	2,614	0.0015	-0.001(3x10^{-4})	0.001	-0.006(0.004)	0.09	-		-		-
			-0.001(3x10^{-4})	0.001	-0.012(0.004)	0.002	0.016(0.004)	2x10^{-4}	1.18	0.55	29.57, 10^{-4}
	39,767	0.05	-6x10^{-4}(6x10^{-5})	<10^{-10}	-0.003 (0.001)	0.003	-		-		-
			-0.001(6x10^{-5})	<10^{-10}	-0.007(0.001)	3x10^{-10}	0.012(0.001)	<10^{-10}	1.10	0.33	211.34, <10^{-10}

Abbreviations: ADHD, Attention-Deficit/Hyperactivity Disorder; ASD, Autism Spectrum Disorder; df, degrees of freedom; iPSYCH, The Lundbeck Foundation Initiative for Integrative Psychiatric Research; LRT, likelihood-ratio test; MVR, multivariable regression; N_{SNPs}, number of SNPs; P_{thr}, *P*-value threshold; woADHD, without ADHD; UVR, univariable regression; ΔR^2, difference in Regression R^2 between MVR and UVR

Estimated ASD effect ($\hat{\theta}_{ASD}$) and ADHD effect ($\hat{\theta}_{*ADHD}$) on general intelligence as fitted with ASD-MVR (Supplementary Figure 1i). Estimated ADHD effect ($\hat{\theta}_{ADHD}$) and ASD effect ($\hat{\theta}_{\#ASD}$) on general intelligence as fitted with ADHD-MVR (Supplementary Figure 1j). Sets of independent ASD (G_i) and ADHD (G_j) genetic variants were selected from ASD(iPSYCH, woADHD) and ADHD(iPSYCH) GWAS statistics respectively and are shown for two *P*-value thresholds (P_{thr}<0.0015, P_{thr}<0.05). SNP estimates for ASD ($\hat{\beta}_{ASD}$), ADHD ($\hat{\beta}_{ADHD}$) and EA ($\hat{\beta}_{Intelligence}$) were extracted from ASD(iPSYCH, woADHD), ADHD(iPSYCH) and intelligence (CTG lab) GWAS statistics respectively. All MVR effects are presented as standard deviation-related changes in general intelligence per increase in log odds of ASD or ADHD liability. Individual effect estimates, standard errors and corresponding *P*-values (t-statistic, two-sided test) are shown. The multiple-testing-adjusted threshold is *P*<0.0125.The model fit of MVRs and UVRs was compared with likelihood-ratio tests (LRTs).

Supplementary Table 16: Specificity analyses related to risk for other psychiatric disorders (independent variable), ASD-MVR (P_{thr}<0.0015; P_{thr}<0.05)

Variant selection			Intercept		ASD effect		Other disorder effect		VIF	MVR to UVR model fit comparison	
G_i	N_{SNPs}	P_{thr}	$\hat{\theta}_{0*}$(SE)	*P*	$\hat{\theta}_{ASD}$(SE)	*P*	$\hat{\theta}_*$(SE)	*P*		ΔR^2 (%)	LRT-χ^2(df=1), *P*
ASD (iPSYCH, woADHD)	2,644	0.0015	0.001(10^{-4})	$3x10^{-5}$	-0.001(0.002)	0.45	-		-		-
			0.001($2x10^{-4}$)	$3x10^{-5}$	-0.001(0.002)	0.55	$\hat{\theta}_{*MDD}$=-0.004(0.006)	0.46	1.04	0.02	1.37, 0.45
	50,904	0.05	$3x10^{-4}$ ($4x10^{-5}$)	<10^{-10}	$7x10^{-4}$($5x10^{-4}$)	0.13	-		-		-
			$3x10^{-4}$($4x10^{-5}$)	<10^{-10}	0.001($5x10^{-4}$)	0.002	$\hat{\theta}_{*MDD}$=-0.012(0.001)	<10^{-10}	1.02	0.29	264.96, <10^{-10}
	1,786	0.0015	0.001($3x10^{-4}$)	0.01	-0.001(0.003)	0.78	-		-		-
			0.001($3x10^{-4}$)	0.02	-0.001(0.003)	0.83	$\hat{\theta}_{*SCZ}$=0.016(0.004)	$3x10^{-4}$	1.00	0.71	37.92, $3x10^{-4}$
	31,026	0.05	$2x10^{-4}$($5x10^{-5}$	$6x10^{-5}$	0.002($8x10^{-4}$)	0.01	-		-		-
			$2x10^{-4}$($5x10^{-5}$)	10^{-4}	0.002(0.001)	0.02	$\hat{\theta}_{*SCZ}$ =0.006(0.001)	$3x10^{-9}$	1.00	0.11	73.31, $3x10^{-9}$
	1,859	0.0015	0.001($2x10^{-4}$)	0.003	-0.001(0.003)	0.61	-		-		-
			0.001($2x10^{-4}$)	0.004	-0.001(0.003)	0.61	$\hat{\theta}_{*BD}$=0.019(0.004)	$6x10^{-7}$	1.00	1.33	71.40, $6x10^{-7}$
	32,367	0.05	$2x10^{-4}$($5x10^{-5}$)	$2x10^{-6}$	0.002($7x10^{-4}$)	0.04	-		-		-
			$2x10^{-4}$($5x10^{-5}$)	$7x10^{-6}$	0.002(0.001)	0.033	$\hat{\theta}_{*BD}$ =0.009(0.001)	<10^{-10}	1.00	0.41	272.24, <10^{-10}

Abbreviations: ASD, Autism Spectrum Disorder; BD, Bipolar Disorder; df, degrees of freedom; iPSYCH, The Lundbeck Foundation Initiative for Integrative Psychiatric Research; LRT, likelihood-ratio test; MDD; Major Depressive Disorder; MVR, multivariable regression; PGC, Psychiatric Genomics Consortium; SCZ, Schizophrenia; P_{thr}, *P*-value threshold; woADHD; without ADHD; UVR, univariable regression; ΔR^2, difference in Regression R^2 between MVR and UVR

Estimated ASD effect ($\hat{\theta}_{ASD}$), MDD ($\hat{\theta}_{*MDD}$), SCZ ($\hat{\theta}_{*SCZ}$) and BD ($\hat{\theta}_{*BD}$) effects on EA as fitted with ASD-MVR (Supplementary Figure 1k, Supplementary Figure 5a). Sets of independent ASD (G_i) genetic variants were selected from ASD(iPSYCH, woADHD) and are shown for two *P*-value thresholds (P_{thr}<0.0015, P_{thr}<0.05). SNP estimates $\hat{\beta}_{ASD,}$ $\hat{\beta}_{MDD}$, $\hat{\beta}_{SCZ,}$ $\hat{\beta}_{BD}$ and $\hat{\beta}_{EA}$ were extracted from ASD(iPSYCH, woADHD), MDD(PGC), SCZ(PGC), BD(PGC) and EA(SSGAC) GWAS statistics, respectively. All MVR effects are presented as change in years of schooling per increase in log odds of ASD or MDD, SCZ or BD liability. Individual effect estimates, standard errors and corresponding *P*-values (t-statistic, two-sided test) are shown. The multiple-testing-adjusted threshold is *P*<0.0042. The model fit of MVRs and UVRs was compared with likelihood-ratio tests (LRTs).

Supplementary Table 17: Specificity analyses related to risk for other psychiatric disorders (independent variable), ADHD-MVR (P_{thr}<0.0015; P_{thr}<0.05)

Variant selection			Intercept		ADHD effect		Other disorder effect		VIF	MVR to UVR model fit comparison	
G_j	N_{SNPs}	P_{thr}	$\hat{\theta}_{0\#}$(SE)	P	$\hat{\theta}_{ADHD}$(SE)	P	$\hat{\theta}_{\#}$(SE)	P		ΔR^2 (%)	LRT-χ^2(df=1), P
ADHD (iPSYCH)	2,567	0.0015	-0.001($2x10^{-4}$)	$<10^{-10}$	-0.005(0.003)	0.11	-		-		-
			-0.001($2x10^{-4}$)	$4x10^{-10}$	-0.002(0.003)	0.56	$\hat{\theta}_{\#MDD}$=-0.033(0.007)	$5x10^{-7}$	1.04	0.98	76.87, $4x10^{-7}$
	38,093	0.05	-0.001($4x10^{-5}$)	$<10^{-10}$	-0.005(0.001)	10^{-10}	-		-		-
			-0.001($4x10^{-5}$)	$<10^{-10}$	-0.004(0.001)	$5x10^{-7}$	$\hat{\theta}_{\#MDD}$=-0.013(0.001)	$<10^{-10}$	1.02	0.23	181.10, $<10^{-10}$
	2,278	0.0015	-0.001($3x10^{-4}$)	$7x10^{-7}$	-0.007(0.004)	0.056	-		-		-
			-0.001($3x10^{-4}$)	$9x10^{-7}$	-0.007(0.004)	0.059	$\hat{\theta}_{\#SCZ}$=-0.006(0.004)	0.14	1.00	0.09	6.91, 0.14
	31,395	0.05	-0.001($5x10^{-5}$)	$<10^{-10}$	-0.007(0.001)	$<10^{-10}$	-		-		-
			-0.001($5x10^{-5}$)	$<10^{-10}$	-0.007(0.001)	$<10^{-10}$	$\hat{\theta}_{\#SCZ}$=0.003(0.001)	0.007	1.00	0.02	16.21, 0.007
	2,340	0.0015	-0.001($2x10^{-4}$)	10^{-7}	-0.007(0.003)	0.040	-		-		-
			-0.001($2x10^{-4}$)	$8x10^{-8}$	-0.007(0.003)	0.042	$\hat{\theta}_{\#BD}$=0.007(0.004)	0.046	1.00	0.17	12.56, 0.046
	32,788	0.05	-0.001($5x10^{-5}$)	$<10^{-10}$	-0.007(0.001)	$<10^{-10}$	-		-		-
			-0.001($5x10^{-5}$)	$<10^{-10}$	-0.007(0.001)	$<10^{-10}$	$\hat{\theta}_{\#BD}$=0.008(0.001)	$<10^{-10}$	1.00	0.29	205.72, $<10^{-10}$

Abbreviations: ADHD, Attention-Deficit/Hyperactivity Disorder; BD, Bipolar Disorder; df, degrees of freedom; iPSYCH, The Lundbeck Foundation Initiative for Integrative Psychiatric Research; LRT, likelihood-ratio test; MDD; Major Depressive Disorder; MVR, multivariable regression; PGC, Psychiatric Genomics Consortium; SCZ, Schizophrenia; P_{thr}, *P*-value threshold; woADHD; without ADHD; UVR, univariable regression; ΔR^2, difference in Regression R^2 between MVR and UVR

Estimated ADHD ($\hat{\theta}_{ADHD}$), MDD ($\hat{\theta}_{\#MDD}$), SCZ ($\hat{\theta}_{\#SCZ}$) and BD ($\hat{\theta}_{\#BD}$) effect on EA as fitted with ADHD-MVR (Supplementary Figure 1l, Supplementary Figure 5b). Sets of independent ADHD (G_j) genetic variants were selected from ADHD(iPSYCH) and are shown for two *P*-value thresholds (P_{thr}<0.0015, P_{thr}<0.05). SNP estimates $\hat{\beta}_{ADHD}$, $\hat{\beta}_{MDD}$, $\hat{\beta}_{SCZ}$, $\hat{\beta}_{BD}$ and $\hat{\beta}_{EA}$ were extracted from ADHD(iPSYCH), MDD(PGC), SCZ(PGC), BD(PGC) and EA(SSGAC) GWAS statistics, respectively. All MVR effects are presented as change in years of schooling per increase in log odds of ADHD or MDD, SCZ or BD liability. Individual effect estimates, standard errors and corresponding *P*-values (t-statistic, two-sided test) are shown. The multiple-testing-adjusted threshold is *P*<0.0042. The model fit of MVRs and UVRs was compared with likelihood-ratio tests (LRTs).

Supplementary Table 18: Genetic correlations of ASD and ADHD with general intelligence

Sample 1	Sample 2	r_g (SE)	*P*
Intelligence(CTG)	ASD(iPSYCH, woADHD)	0.25(0.04)	$<10^{-10}$
	ASD(PGC)	0.19(0.04)	$2x10^{-7}$
	ADHD(iPSYCH)	-0.33(0.03)	$<10^{-10}$

Abbreviations: ADHD, Attention-Deficit/Hyperactivity Disorder; ASD, Autism Spectrum Disorder; CTG, Complex Trait Genetics lab; iPSYCH, The Lundbeck Foundation Initiative for Integrative Psychiatric Research; PGC, Psychiatric Genomics Consortium; rg, genetic correlation; woADHD, without ADHD;

Genetic correlations (r_g) with intelligence were estimated using summary statistics and unconstrained LD score correlation[1]. Individual genetic correlation estimates, standard errors and corresponding *P*-values (Z-statistic, two-sided test) are shown. The multiple testing threshold was *P*<0.002.

Supplementary Table 19: SNP-heritability estimates

Phenotype	Sample	SNP-h^2 (SE)	λ_{GC}	Intercept (SE)
ASD	ASD(iPSYCH, woADHD)	0.13(0.01)	1.13	1.01(0.01)
	ASD(PGC)	0.26(0.03)	1.05	0.97(0.01)
ADHD	ADHD(iPSYCH)	0.26(0.02)	1.23	1.03(0.01)
MDD	MDD(PGC)	0.095(0.01)	1.24	0.99(0.01)
SCZ	SCZ(PGC)	0.24(0.01)	1.50	1.05(0.01)
BD	BD(PGC)	0.18(0.01)	1.27	1.02(0.01)
Years of schooling	EA(SSGAC)	0.11(0.003)	2.10	1.03(0.01)
General intelligence	Intelligence(CTG)	0.18(0.006)	1.75	1.08(0.01)

Abbreviations: ADHD, Attention-Deficit/Hyperactivity Disorder; ASD, Autism Spectrum Disorder; BD, Bipolar Disorder; EA; educational attainment; CTG, Complex Trait Genetics lab; iPSYCH, The Lundbeck Foundation Initiative for Integrative Psychiatric Research; MDD, Major Depressive Disorder; PGC, Psychiatric Genomics Consortium; SCZ, Schizophrenia; SSGAC, Social Science Genetic Association Consortium; λ_{GC}, lambda GC; woADHD, without ADHD

SNP-heritability (SNP-h^2) was estimated with LD score regression analysis[8]. SNP-h^2 estimates for EA and general intelligence were calculated on the observed scale and for psychiatric disorders on a liability scale assuming a population prevalence of 0.012 (ASD), 0.05 (ADHD), 0.162 (MDD), 0.007 (SCZ) and 0.006 (BD).

Supplementary Figures

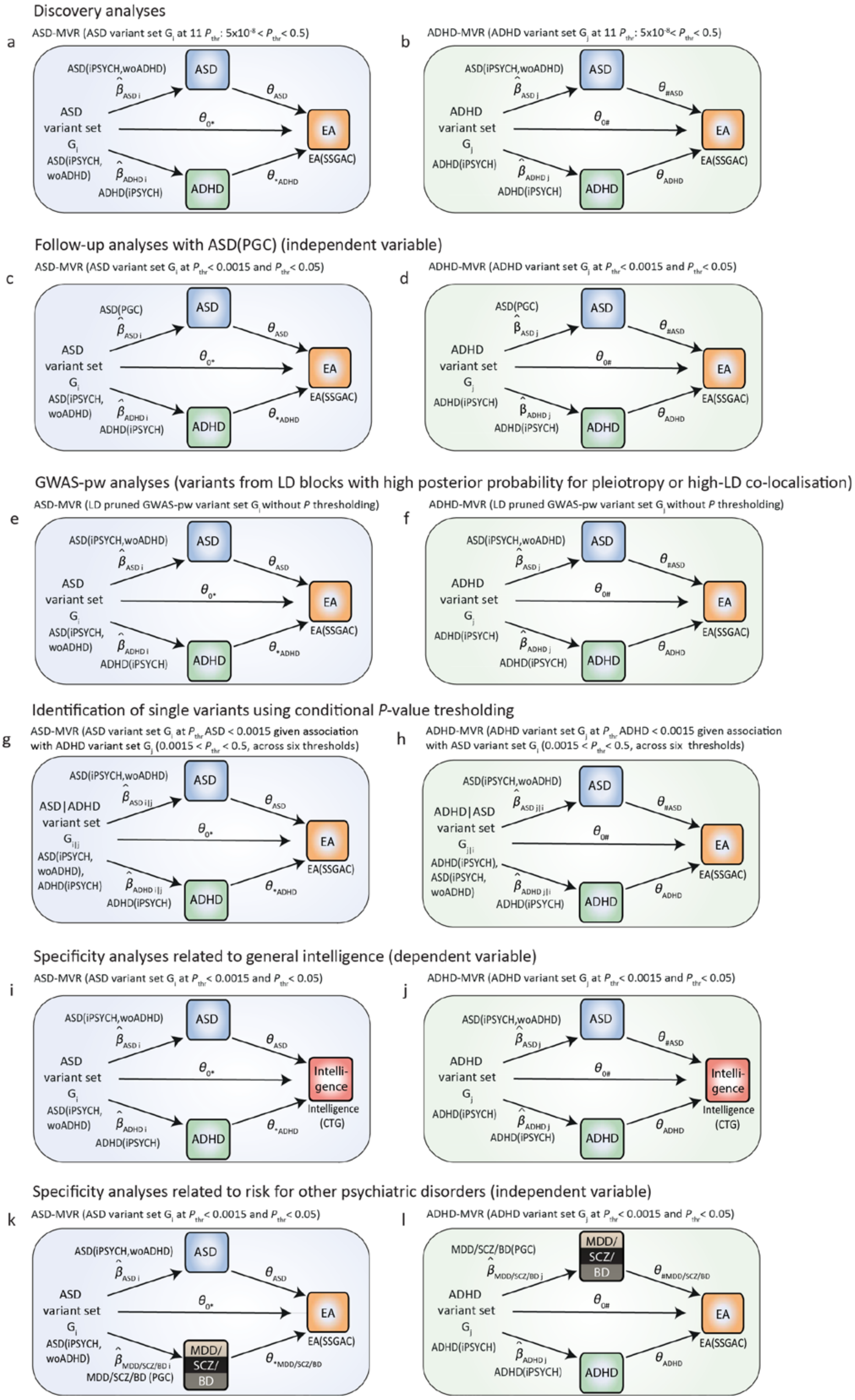

Discovery analyses
a
ASD-MVR (ASD variant set G_i at 11 P_thr: 5x10^-8 < P_thr < 0.5)
ASD(iPSYCH,woADHD)
ASD
ASD variant set G_i
ASD(iPSYCH, woADHD)
ADHD(iPSYCH)
ADHD
EA
EA(SSGAC)
b
ADHD-MVR (ADHD variant set G_j at 11 P_thr: 5x10^-8 < P_thr < 0.5)
ASD(iPSYCH,woADHD)
ADHD variant set G_j
ADHD(iPSYCH)
EA(SSGAC)
Follow-up analyses with ASD(PGC) (independent variable)
c
ASD-MVR (ASD variant set G_i at P_thr < 0.0015 and P_thr < 0.05)
ASD(PGC)
ASD variant set G_i
ASD(iPSYCH, woADHD)
ADHD(iPSYCH)
EA(SSGAC)
d
ADHD-MVR (ADHD variant set G_j at P_thr < 0.0015 and P_thr < 0.05)
ASD(PGC)
ADHD variant set G_j
ADHD(iPSYCH)
EA(SSGAC)
GWAS-pw analyses (variants from LD blocks with high posterior probability for pleiotropy or high-LD co-localisation)
e
ASD-MVR (LD pruned GWAS-pw variant set G_i without P thresholding)
ASD(iPSYCH,woADHD)
ASD variant set G_i
ASD(iPSYCH, woADHD)
ADHD(iPSYCH)
EA(SSGAC)
f
ADHD-MVR (LD pruned GWAS-pw variant set G_j without P thresholding)
ASD(iPSYCH,woADHD)
ADHD variant set G_j
ADHD(iPSYCH)
EA(SSGAC)
Identification of single variants using conditional P-value tresholding
g
ASD-MVR (ASD variant set G_i at P_thr ASD < 0.0015 given association with ADHD variant set G_j (0.0015 < P_thr < 0.5, across six thresholds)
ASD(iPSYCH,woADHD)
ASD|ADHD variant set G_i|j
ASD(iPSYCH, woADHD), ADHD(iPSYCH)
ADHD(iPSYCH)
EA(SSGAC)
h
ADHD-MVR (ADHD variant set G_j at P_thr ADHD < 0.0015 given association with ASD variant set G_i (0.0015 < P_thr < 0.5, across six thresholds)
ASD(iPSYCH,woADHD)
ADHD|ASD variant set G_j|i
ADHD(iPSYCH), ASD(iPSYCH, woADHD)
ADHD(iPSYCH)
EA(SSGAC)
Specificity analyses related to general intelligence (dependent variable)
i
ASD-MVR (ASD variant set G_i at P_thr < 0.0015 and P_thr < 0.05)
ASD(iPSYCH,woADHD)
ASD variant set G_i
ASD(iPSYCH, woADHD)
ADHD(iPSYCH)
Intelli-gence
Intelligence (CTG)
j
ADHD-MVR (ADHD variant set G_j at P_thr < 0.0015 and P_thr < 0.05)
ASD(iPSYCH,woADHD)
ADHD variant set G_j
ADHD(iPSYCH)
Intelli-gence
Intelligence (CTG)
Specificity analyses related to risk for other psychiatric disorders (independent variable)
k
ASD-MVR (ASD variant set G_i at P_thr < 0.0015 and P_thr < 0.05)
ASD(iPSYCH,woADHD)
ASD variant set G_i
ASD(iPSYCH, woADHD)
MDD/SCZ/BD (PGC)
MDD/ SCZ/ BD
EA(SSGAC)
l
ADHD-MVR (ADHD variant set G_j at P_thr < 0.0015 and P_thr < 0.05)
MDD/SCZ/BD(PGC)
MDD/ SCZ/ BD
ADHD variant set G_j
ADHD(iPSYCH)
ADHD(iPSYCH)
EA(SSGAC)

Supplementary Figure 1: MVR study design

Acyclic graphs illustrating the multivariable regression (MVR) design applied at different stages of the study detailing GWAS statistics for both, variant set selection and SNP estimate ($\hat{\beta}$) extraction. For ASD-MVR, a set of ASD-related variants (G_i) and for ADHD MVR, a set of ADHD-related variants (G_j) was selected at different *P*-value thresholds (P_{thr}) as shown for **(a-b)** discovery analyses, **(c-d)** follow-up analyses, **(e-f)** gwas-pw analyses, **(g-h)** the identification of single variants with conditional *P*-value thresholding analyses (using subsets of G_i and G_j, i.e. $G_{i|j}$ and $G_{j|i}$) and **(i-l)** specificity analyses, including **(i-j)** general intelligence as dependent variable and **(k-l)** risk for other psychiatric disorder (MDD, SCZ, BD) as independent variables. For GWAS-pw analyses **(e-f)**, ASD-related and ADHD-related variants were selected from linkage disequilibrium (LD) blocks with high posterior probability (>0.9) for pleiotropy or, equivalently, high-LD co-localisation; variants within high-confidence regions were pruned for LD (LD-r^2<0.25 within ±500 kb) without applying *P*-value thresholding. MVR effects are denoted by $\hat{\theta}$ and MVR intercepts by $\hat{\theta}_0$. "*" Estimation of polygenic risk effects using alleles that were selected to increase liability for ASD, but are shared, by position, with risk alleles for other disorders; "#" Estimation of polygenic risk effects using alleles that were selected to increase liability for ADHD, but are shared, by position, with risk alleles for other disorders.

Abbreviations: ADHD, Attention-Deficit/Hyperactivity Disorder; ASD, Autism Spectrum Disorder; BD, Bipolar Disorder; EA, Educational attainment, GWAS, genome-wide association study, iPSYCH, The Lundbeck Foundation Initiative for Integrative Psychiatric Research; MDD; Major Depressive Disorder; MVR, multivariable regression; P_{thr}, *P*-value threshold; PGC, Psychiatric Genomics Consortium; SCZ, Schizophrenia; SSGAC, Social Science Genetic Consortium; woADHD; without ADHD

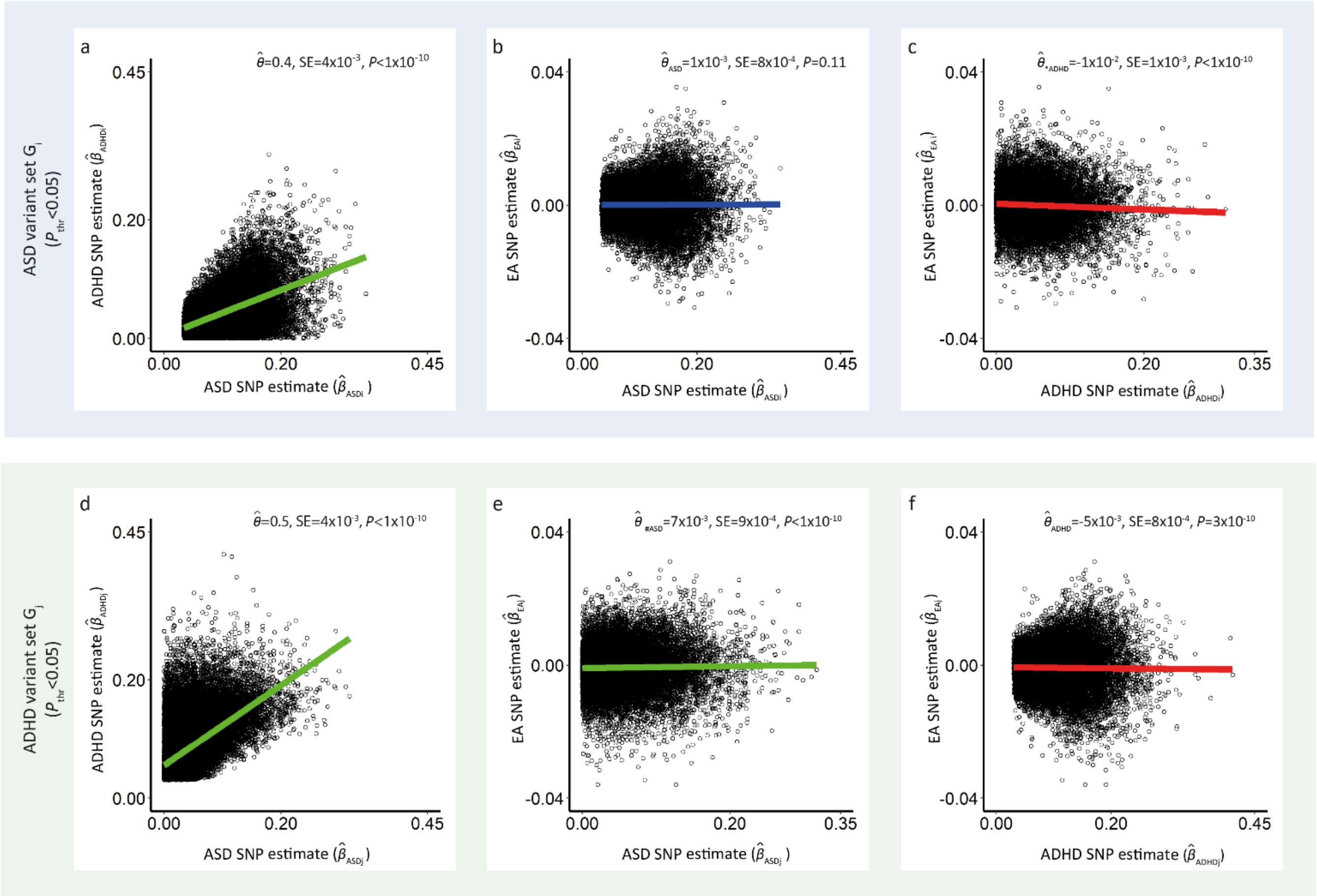

Supplementary Figure 2: Bivariate analyses of ASD, ADHD and EA genetic effects using ASD and ADHD concordant variant sets

Sets of independent ASD-related (G_i) and ADHD-related (G_j) genetic variants were selected from ASD(iPSYCH, woADHD) and ADHD(iPSYCH) GWAS statistics respectively (P_{thr}<0.05). SNP estimates for ASD ($\hat{\beta}_{ASD}$), ADHD ($\hat{\beta}_{ADHD}$) and EA ($\hat{\beta}_{EA}$) were extracted from ASD(iPSYCH, woADHD; N=32,985) and ADHD(iPSYCH; N=37,076) and EA(SSGAC; N=766,345) GWAS statistics respectively. Only genetic variants with the same risk-increasing allele for both ASD and ADHD (concordant variants) were included. **(a-c)** Using univariable weighted regression models for ASD variants (G_i), **(a)** ADHD SNP estimates ($\hat{\beta}_{ADHD}$) were regressed on ASD SNP estimates ($\hat{\beta}_{ASD}$), **(b)** EA SNP estimates ($\hat{\beta}_{EA}$) were regressed on ASD SNP estimates ($\hat{\beta}_{ASD}$), and **(c)** EA SNP estimates ($\hat{\beta}_{EA}$) were regressed on ADHD SNP estimates ($\hat{\beta}_{ADHD}$). **(d-f)** Using univariable weighted regression models and ADHD variants (G_j), **(d)** ADHD SNP estimates ($\hat{\beta}_{ADHD}$) were regressed on ASD SNP estimates ($\hat{\beta}_{ASD}$), **(e)** EA SNP estimates ($\hat{\beta}_{EA}$) were regressed on ASD SNP estimates ($\hat{\beta}_{ASD}$), and **(f)** EA SNP estimates ($\hat{\beta}_{EA}$) were regressed on ADHD SNP estimates ($\hat{\beta}_{ADHD}$). All regressions allowed for an intercept. A green regression line denotes a positive relationship between SNP estimates, a blue regression line reflects no significant relationship and a red line indicates a negative relationship. Regression estimates ($\hat{\theta}$), corresponding standard errors (SE) and *P*-values are shown for each regression model. Source data are provided as a Source Data file.

Abbreviations: ASD, Autism Spectrum Disorder; ADHD, Attention-Deficit/Hyperactivity Disorder; EA, educational attainment; P_{thr}, *P*-value threshold; SNP, single-nucleotide polymorphism

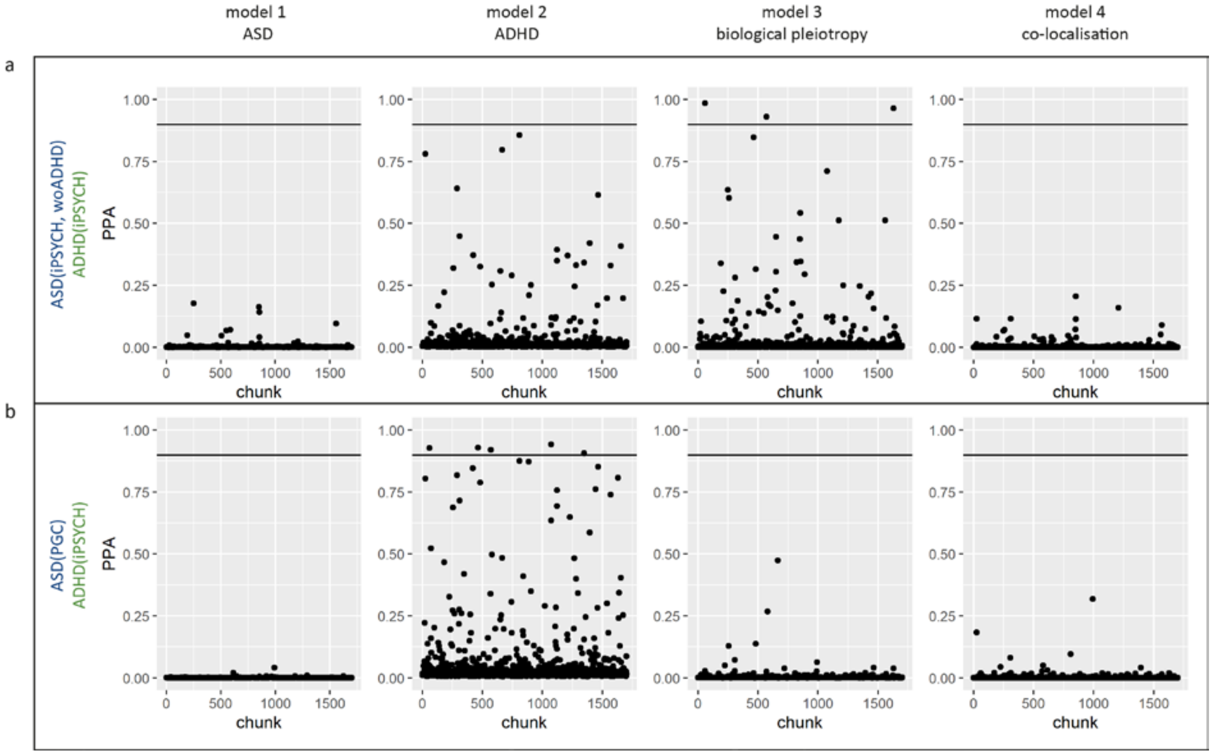

Supplementary Figure 3: Evidence for pleiotropy and co-localisation using gwas-pw

gwas-pw analyses[9] were carried out using GWAS summary statistics without applying *P*-value selection criteria for **(a)** ASD(iPSYCH,woADHD; N=32,985) and ADHD(iPSYCH; N=37,076) summary statistics, and **(b)** ASD(PGC; N=10,610) and ADHD(iPSYCH; N=37,076) summary statistics. After dividing the genome into approximately independent LD blocks, gwas-pw estimates the posterior probability that a given LD block contains a genetic variant associated with ASD (model 1), a genetic variant associated with ADHD (model 2), a genetic variant associated with both disorders, reflecting biological pleiotropy or high-LD co-localisation (model 3), or multiple genetic variants that are each associated with a different disorder (co-localisation in the presence of low/moderate LD, model 4). Evidence for model 3 and model 4 was evaluated at a stringent posterior probability threshold (>0.9), indicated by a black horizontal line in each plot. Source data are provided as a Source Data file.

Abbreviations: ADHD, Attention-Deficit/Hyperactivity Disorder; ASD, Autism Spectrum Disorder; iPSYCH, The Lundbeck Foundation Initiative for Integrative Psychiatric Research; PGC, Psychiatric Genomics Consortium; PPA, posterior probability; woADHD, without ADHD

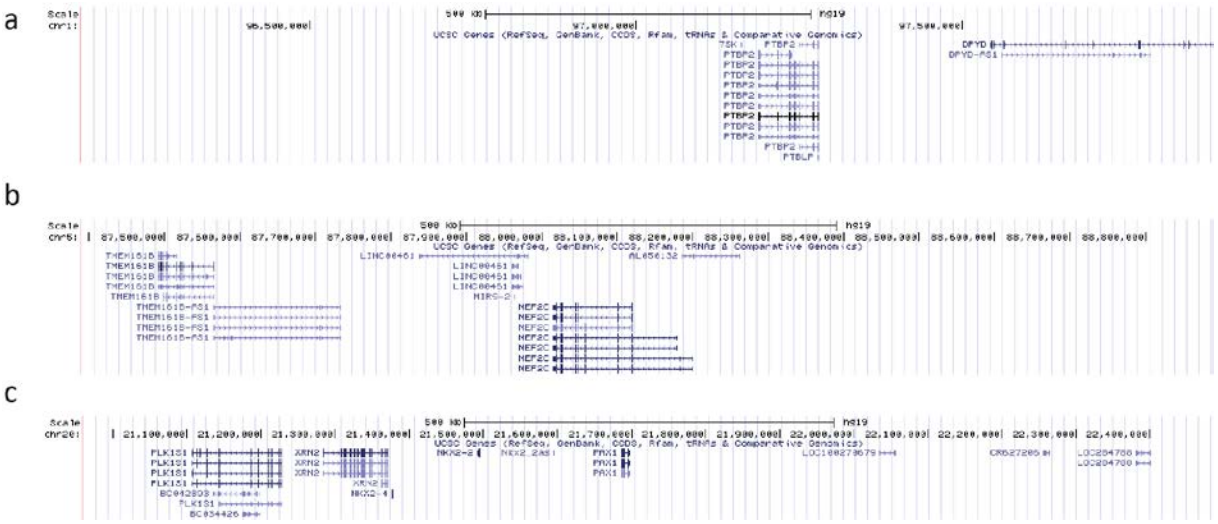

Supplementary Figure 4: Genes annotated within pleiotropic genomic segments

Approximately independent LD blocks with evidence for pleiotropy and/or co-localisation (posterior probability > 0.9), were identified using gwas-pw analyses[9] and summary statistics for ASD(iPSYCH,woADHD) and ADHD(iPSYCH). Genes annotated within these regions were identified using UCSC genome browser[10]. **(a)** chromosome 1: 96151539-97885200 bp, **(b)** chromosome 5: 87391024-88890628 bp, **(c)** chromosome 20: 20961267-22485526 bp.

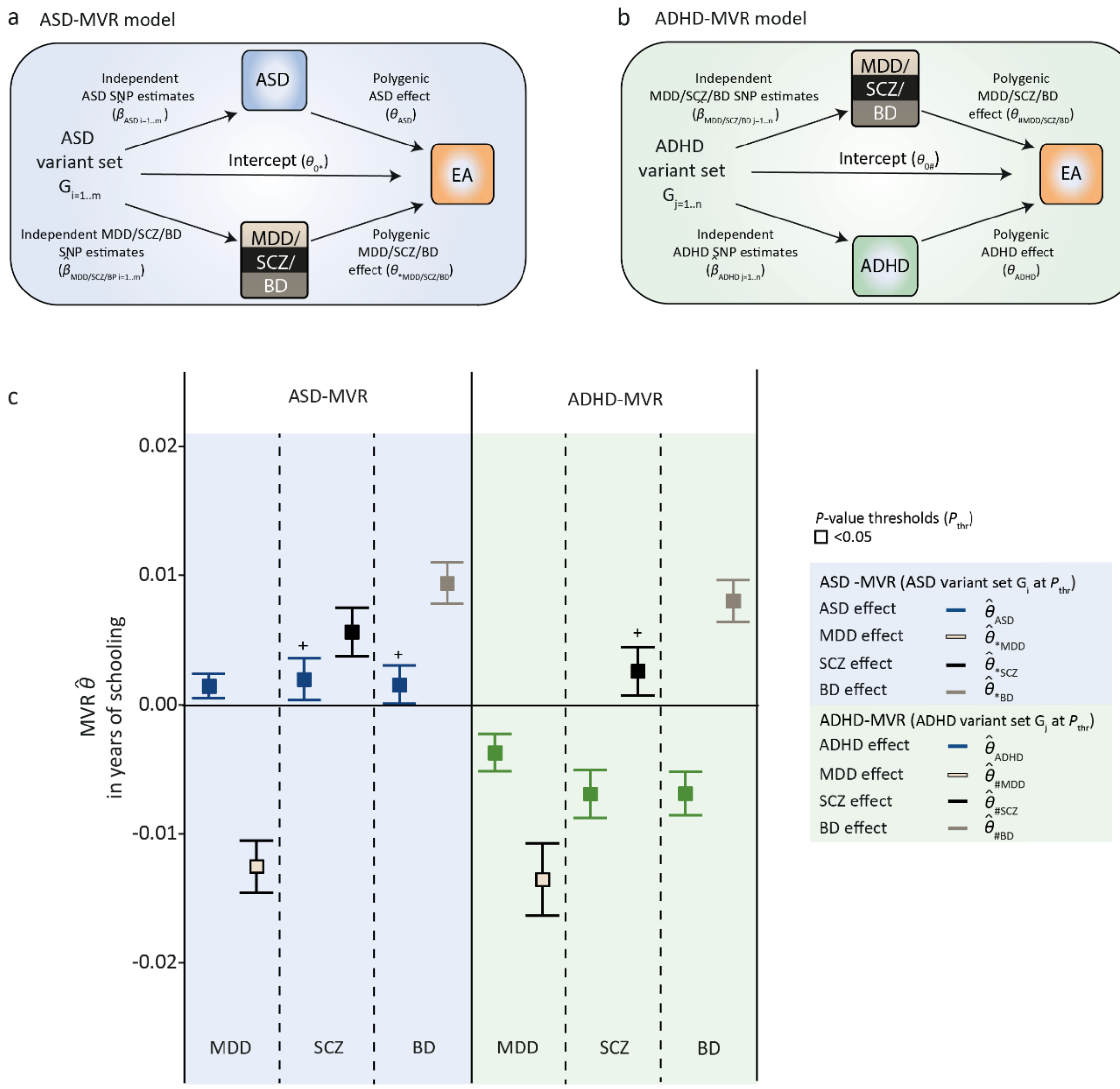

Supplementary Figure 5: Cross-disorder associations with educational attainment for other disorders

Acyclic graphs illustrating the multivariable regression (MVR) design for **(a)** a set of independent ASD-related variants G_i (ASD-MVR) and **(b)** a set of independent ADHD-related variants G_j (ADHD-MVR). For ASD-MVR **(a)**, ASD-increasing marker alleles (G_i) were aligned to ASD ($\hat{\beta}_{ASD}$), MDD ($\hat{\beta}_{MDD}$), SCZ ($\hat{\beta}_{SCZ}$), BD ($\hat{\beta}_{BD}$) and EA ($\hat{\beta}_{EA}$), capturing genetic association at the single variant level. Using the MVR framework, the aggregate association effect with EA across all variants was simultaneously estimated for ASD risk (θ_{ASD}) and other disorders (θ_{*MDD}, θ_{*SCZ} and θ_{*BD}), including a regression intercept (θ_{0*}). * Estimation of polygenic risk effects using alleles that were selected to increase liability for ASD, but are shared, by position, with risk for other disorders. **(b)** Likewise, for ADHD-MVR, increaser marker alleles (G_j) were aligned to ADHD ($\hat{\beta}_{ADHD}$), MDD ($\hat{\beta}_{MDD}$), SCZ ($\hat{\beta}_{SCZ}$), BD ($\hat{\beta}_{BD}$) and EA ($\hat{\beta}_{EA}$) GWAS SNP estimates and aggregate association effect with EA across all variants simultaneously estimated for ADHD risk (θ_{ADHD}) and other disorders ($\theta_{\#MDD}$, $\theta_{\#SCZ}$ and $\theta_{\#BD}$), including a regression intercept ($\theta_{0\#}$). # Estimation of polygenic risk effects using alleles that were selected to increase liability for ADHD, but are shared, by position, with risk for other disorders. **(c)** ASD-MVR ($\hat{\theta}_{ASD}$; $\hat{\theta}_{*MDD}$; $\hat{\theta}_{*SCZ}$; $\hat{\theta}_{*BD}$) and ADHD-MVR ($\hat{\theta}_{ADHD}$; $\hat{\theta}_{\#MDD}$; $\hat{\theta}_{\#SCZ}$; $\hat{\theta}_{\#BD}$) effects as change in years of schooling per increase in log odds of ASD or ADHD liability. Multivariate inverse-variance-weighted regression estimates and corresponding 95% confidence intervals (bars) are shown. Individual effect estimates, standard errors and corresponding *P*-values (t-statistic, two-sided test) are provided in Supplementary Table 16-17. All effects passed the multiple testing threshold of $P<0.0042$, unless indicated otherwise (+).Sets of independent ASD (G_i) and ADHD (G_j) genetic variants were selected from ASD(iPSYCH, woADHD) and ADHD(iPSYCH) GWAS statistics respectively and are shown for *P*-value threshold ($P_{thr}<0.05$). SNP estimates $\hat{\beta}_{ASD}$, $\hat{\beta}_{ADHD}$, $\hat{\beta}_{MDD}$, $\hat{\beta}_{SCZ}$, $\hat{\beta}_{BD}$ and $\hat{\beta}_{EA}$ were extracted from ASD(iPSYCH, woADHD; N=32,985), ADHD(iPSYCH; N=37,076), MDD(PGC; N=173,005), SCZ(PGC; N=65,967), BD(PGC; N=41,653) and EA(SSGAC; N=766,345) GWAS statistics respectively. Source data are provided as a Source Data file.

Abbreviations: ADHD, Attention-Deficit/Hyperactivity Disorder; ASD, Autism Spectrum Disorder; BD, Bipolar Disorder; EA, Educational attainment, iPSYCH, The Lundbeck Foundation Initiative for Integrative Psychiatric Research; MDD; Major

Depressive Disorder; MVR, multivariable regression; P_{thr}, *P*-value threshold; PGC, Psychiatric Genomics Consortium; SCZ, Schizophrenia; SSGAC, Social Science Genetic Consortium; woADHD; without ADHD

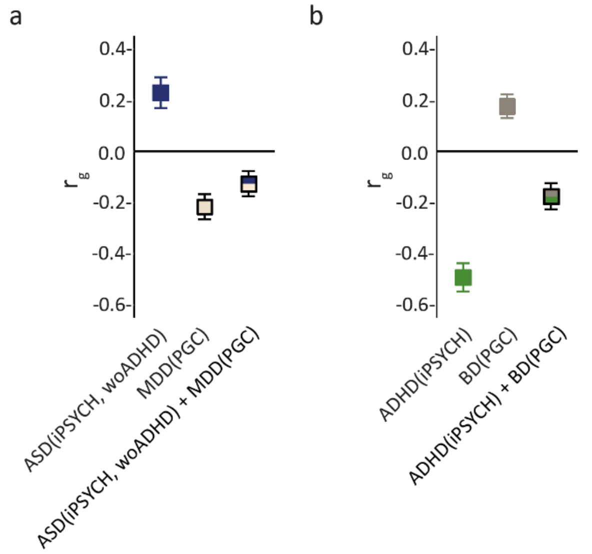

Supplementary Figure 6: Genetic correlations with EA for cross-disorder meta-analyses

Genetic correlations (r_g) of educational attainment (EA) with ASD, ADHD, BD, MDD, and combined ASD + MDD risk as well as combined ADHD + BD risk were estimated using unconstrained LD score correlation[1]. Genetic correlations with EA(SSGAC; N=766,345) were estimated for **(a)** ASD(iPSYCH, woADHD; N=32.985), MDD(PGC; N=173,005) and a combination of these summary statistics (cross-disorder meta-analysis), and, analogously, for **(b)** for ADHD(iPSYCH; N=37,076), BD(PGC; N=41,653) and a combination of these summary statistics (cross-disorder meta-analysis). Cross-disorder meta-analyses were conducted with METACARPA, allowing for sample overlap[11]. LDSC correlation estimates with 95% confidence intervals (bars) are shown. Individual genetic correlation estimates, standard errors and corresponding *P*-values (Z-statistic, two-sided test) are provided in Supplementary Table 6. All tests passed the multiple testing threshold of *P*<0.002. Source data are provided as a Source Data file.

Abbreviations: ADHD, Attention-Deficit/Hyperactivity Disorder; ASD, Autism Spectrum Disorder; BD, Bipolar Disorder; MDD, Major Depressive Disorder; iPSYCH, The Lundbeck Foundation Initiative for Integrative Psychiatric Research; PGC, Psychiatric Genetics Consortium; r_g, genetic correlation; SSGAC, Social Science Genetic Association Consortium; woADHD, without ADHD

Supplementary References

1. Bulik-Sullivan B, Finucane HK, Anttila V, et al. An atlas of genetic correlations across human diseases and traits. *Nat Genet*. 2015;47:1236–1241. doi:10.1038/ng.3406

2. Pickrell JK, Berisa T, Liu JZ, Ségurel L, Tung JY, Hinds DA. Detection and interpretation of shared genetic influences on 42 human traits. *Nat Genet*. 2016;48(7):709-717. doi:10.1038/ng.3570

3. Leeuw CA de, Mooij JM, Heskes T, Posthuma D. MAGMA: Generalized Gene-Set Analysis of GWAS Data. *PLOS Computational Biology*. 2015;11(4):e1004219. doi:10.1371/journal.pcbi.1004219

4. Watanabe K, Taskesen E, Bochoven A van, Posthuma D. Functional mapping and annotation of genetic associations with FUMA. *Nature Communications*. 2017;8(1):1826. doi:10.1038/s41467-017-01261-5

5. Wray NR, Ripke S, Mattheisen M, et al. Genome-wide association analyses identify 44 risk variants and refine the genetic architecture of major depression. *Nature Genetics*. 2018;50(5):668. doi:10.1038/s41588-018-0090-3

6. Ruderfer DM, Ripke S, McQuillin A, et al. Genomic Dissection of Bipolar Disorder and Schizophrenia, Including 28 Subphenotypes. *Cell*. 2018;173(7):1705-1715.e16. doi:10.1016/j.cell.2018.05.046

7. Savage JE, Jansen PR, Stringer S, et al. Genome-wide association meta-analysis in 269,867 individuals identifies new genetic and functional links to intelligence. *Nature Genetics*. 2018;50(7):912-919. doi:10.1038/s41588-018-0152-6

8. Bulik-Sullivan BK, Loh P-R, Finucane HK, et al. LD Score regression distinguishes confounding from polygenicity in genome-wide association studies. *Nat Genet*. 2015;47(3):291-295. doi:10.1038/ng.3211

9. Pickrell JK, Berisa T, Liu JZ, Ségurel L, Tung JY, Hinds DA. Detection and interpretation of shared genetic influences on 42 human traits. *Nat Genet*. 2016;48(7):709-717. doi:10.1038/ng.3570

10. Kent WJ, Sugnet CW, Furey TS, et al. The Human Genome Browser at UCSC. *Genome Res*. 2002;12(6):996-1006. doi:10.1101/gr.229102

11. Southam L, Gilly A, Süveges D, et al. Whole genome sequencing and imputation in isolated populations identify genetic associations with medically-relevant complex traits. *Nature Communications*. 2017;8(1):15606. doi:10.1038/ncomms15606
