# Supplementary material for: Discordant associations of educational attainment with ASD and ADHD implicate a polygenic form of pleiotropy
Source: Nat Commun. 2021 Nov 11;12:6534. doi: 10.1038/s41467-021-26755-1 (PMC8586371; doi:10.1038/s41467-021-26755-1)
Supplement: Supplementary file 2 — Description of Additional Supplementary Files [file 41467_2021_26755_MOESM2_ESM.pdf]

Description of Additional Supplementary Files

File Name: Supplementary Data 1
Description: Abbreviations: ADHD, Attention-Deficit/Hyperactivity Disorder; ASD, Autism Spectrum Disorder; iPSYCH, The Lundbeck Foundation Initiative for Integrative Psychiatric Research; P_{thr}, *P*-value threshold; woADHD, without ADHD

Variants were identified as tagged, if SNPs were located within 500kb and LD-$r^2 \geq 0.6$ (listed within the same row). All SNP estimates were aligned according to allele A1. SNP estimates with corresponding standard errors and *P*-values were extracted from ASD(iPSYCH, woADHD) and ADHD(iPSYCH) GWAS summary statistics.

File Name: Supplementary Data 1
Description: Abbreviations: ADHD, Attention-Deficit/Hyperactivity Disorder; ASD, Autism Spectrum Disorder; EA, educational attainment; FRQ, frequency; GWAS, genome-wide association study; HDL; High-Density-Lipoprotein; SNP, Single-Nucleotide Polymorphism; PGC, Psychiatric Genetics Consortium

Characteristics of the 83 loci that were identified at a joint *P*-value threshold of 0.0015 in ASD(iPSYCH, woADHD) and ADHD(iPSYCH) GWAS summary statistics. SNPs depicted in black (N=30) were identical for ASD-MVR and ADHD-MVR, SNPs corresponding to the same index in blue (ASD-MVR) and green (ADHD-MVR) were in high LD > 0.6 (N=53). SNPs depicted in bold reside within the three high-confidence genomic regions identified with gwas-pw. Allele frequencies (FRQ) were extracted from the Haplotype Reference Consortium r1.1. Mapped genes were identified with PLINK software based on RefSeq (build 37) with a 0 kb window around gene boundaries. GWAS hits ($P<5x10^{-8}$), as recorded in the GWAS Catalog, were identified using the UCSC Genome Browser data integrator tool (build 37) (https://genome.ucsc.edu/cgi-bin/hgIntegrator).
