# Supplementary material for: Discordant associations of educational attainment with ASD and ADHD implicate a polygenic form of pleiotropy
Source: Nat Commun. 2021 Nov 11;12:6534. doi: 10.1038/s41467-021-26755-1 (PMC8586371; doi:10.1038/s41467-021-26755-1)
Supplement: Supplementary file 5 — Reporting Summary [file 41467_2021_26755_MOESM5_ESM.pdf]

nature research

Corresponding author(s): Dr. Beate St Pourcain

Last updated by author(s): Aug 12, 2021

Reporting Summary

Nature Research wishes to improve the reproducibility of the work that we publish. This form provides structure for consistency and transparency in reporting. For further information on Nature Research policies, see Authors & Referees and the Editorial Policy Checklist.

Statistics

For all statistical analyses, confirm that the following items are present in the figure legend, table legend, main text, or Methods section.

n/a	Confirmed	
☐	☒	The exact sample size (n) for each experimental group/condition, given as a discrete number and unit of measurement
☐	☒	A statement on whether measurements were taken from distinct samples or whether the same sample was measured repeatedly
☐	☒	The statistical test(s) used AND whether they are one- or two-sided<br>*Only common tests should be described solely by name; describe more complex techniques in the Methods section.*
☐	☒	A description of all covariates tested
☐	☒	A description of any assumptions or corrections, such as tests of normality and adjustment for multiple comparisons
☐	☒	A full description of the statistical parameters including central tendency (e.g. means) or other basic estimates (e.g. regression coefficient) AND variation (e.g. standard deviation) or associated estimates of uncertainty (e.g. confidence intervals)
☐	☒	For null hypothesis testing, the test statistic (e.g. F, t, r) with confidence intervals, effect sizes, degrees of freedom and P value noted<br>*Give P values as exact values whenever suitable.*
☒	☐	For Bayesian analysis, information on the choice of priors and Markov chain Monte Carlo settings
☒	☐	For hierarchical and complex designs, identification of the appropriate level for tests and full reporting of outcomes
☐	☒	Estimates of effect sizes (e.g. Cohen's d, Pearson's r), indicating how they were calculated

Our web collection on statistics for biologists contains articles on many of the points above.

Software and code

Policy information about availability of computer code

Data collection	No software was used for data collection as this study does not include new data collection.
Data analysis	Linkage Disequilibrium SCore (LDSC, v1.0.0) regression and correlation, R:stats library (Rv3.5.1), R:car library (Rv3.5.1), gwas-pw (v0.21), PLINK (v1.90b3w), UCSC Genome Browser data integrator tool (build 37), MAGMA(v1.08) as implemented within FUMA (v1.3.6a). Detailed information about regression equations applied is given in the Methods.

For manuscripts utilizing custom algorithms or software that are central to the research but not yet described in published literature, software must be made available to editors/reviewers. We strongly encourage code deposition in a community repository (e.g. GitHub). See the Nature Research guidelines for submitting code & software for further information.

Data

Policy information about availability of data

All manuscripts must include a data availability statement. This statement should provide the following information, where applicable:

- Accession codes, unique identifiers, or web links for publicly available datasets
- A list of figures that have associated raw data
- A description of any restrictions on data availability

Genome-wide association summary statistics on ASD(PGC), EA(SSGAC), Intelligence(CTG), MDD(PGC), SCZ(PGC) and BD(PGC) are publicly available. Download links and/or respective references are provided in the methods section and data availability statement. Restrictions apply to the availability of summary statistics from the iPSYCH sample. For a description of the restrictions and how to get access to these data, please refer to the data availability statement.

Field-specific reporting

Please select the one below that is the best fit for your research. If you are not sure, read the appropriate sections before making your selection.

☒ Life sciences ☐ Behavioural & social sciences ☐ Ecological, evolutionary & environmental sciences

For a reference copy of the document with all sections, see nature.com/documents/nr-reporting-summary-flat.pdf

Life sciences study design

All studies must disclose on these points even when the disclosure is negative.

Sample size	Sample sizes were determined by the publicly available studies at the time. For educational attainment, general intelligence, major depressive disorder, bipolar disorder and schizophrenia publicly available genome-wide association study (GWAS) summary statistics were retrieved based on the largest European samples available at the time. GWAS summary statistics on Autism Spectrum Disorder and Attention-Deficit/Hyperactivity Disorder were obtained from the iPSYCH consortium, excluding overlapping cases. Independent GWAS summary statistics on Autism Spectrum Disorder were retrieved from the PGC consortium.
Data exclusions	Participants were all of European ancestry. For the Autism Spectrum Disorder sample obtained from iPSYCH, cases with an additional diagnosis for Attention-Deficit/Hyperactivity Disorder were excluded. These criteria were pre-established.
Replication	The reproducibility of the analyses was examined by including two independent Autism Spectrum Disorder samples. Findings were replicated at the $P<0.05$ threshold, but not at the $P<0.0015$ threshold. This might be due to lower power of the PGC data and/or heterogeneity between PGC and iPSYCH samples.
Randomization	Not applicable as we did not study experimental groups.
Blinding	Not applicable as we did not study experimental groups.

Reporting for specific materials, systems and methods

We require information from authors about some types of materials, experimental systems and methods used in many studies. Here, indicate whether each material, system or method listed is relevant to your study. If you are not sure if a list item applies to your research, read the appropriate section before selecting a response.

Materials & experimental systems

n/a	Involved in the study	
☒	☐	Antibodies
☒	☐	Eukaryotic cell lines
☒	☐	Palaeontology
☒	☐	Animals and other organisms
☒	☐	Human research participants
☒	☐	Clinical data

Methods

n/a	Involved in the study	
☒	☐	ChIP-seq
☒	☐	Flow cytometry
☒	☐	MRI-based neuroimaging
